# Spatial distribution of *Mycobacterium tuberculosis* mRNA and secreted antigens in acid-fast negative human antemortem and resected tissue

**DOI:** 10.1016/j.ebiom.2024.105196

**Published:** 2024-06-15

**Authors:** Kievershen Nargan, Joel N. Glasgow, Sajid Nadeem, Threnesan Naidoo, Gordon Wells, Robert L. Hunter, Anneka Hutton, Kapongo Lumamba, Mpumelelo Msimang, Paul V. Benson, Adrie J.C. Steyn

**Affiliations:** a Africa Health Research Institute, University of KwaZulu-Natal, Durban, South Africa; b Department of Microbiology, University of Alabama at Birmingham, Birmingham, AL, USA; c Department of Forensic and Legal Medicine, Walter Sisulu University, Mthatha, South Africa; d Department of Pathology and Laboratory Medicine, University of Texas Health Sciences Center at Houston, Houston, TX, USA; e Division of Pulmonary, Allergy, and Critical Care Medicine, Department of Medicine, University of Alabama at Birmingham, Birmingham, AL, USA; f Department of Anatomical Pathology, National Health Laboratory Service, IALCH, Durban, South Africa; g Department of Pathology, University of Alabama at Birmingham, Birmingham, AL, USA; h Centers for AIDS Research and Free Radical Biology, University of Alabama at Birmingham, Birmingham, AL, USA

**Keywords:** Tuberculosis, Antigen, Ziehl-Neelsen, RNAscope, Diagnosis

## Abstract

**Background:**

The ability to detect evidence of *Mycobacterium tuberculosis* (*Mtb*) infection within human tissues is critical to the study of *Mtb* physiology, tropism, and spatial distribution within TB lesions. The capacity of the widely-used Ziehl-Neelsen (ZN) staining method for identifying *Mtb* acid-fast bacilli (AFB) in tissue is highly variable, which can limit detection of *Mtb* bacilli for research and diagnostic purposes. Here, we sought to circumvent these limitations via detection of *Mtb* mRNA and secreted antigens in human tuberculous tissue.

**Methods:**

We adapted RNAscope, an RNA *in situ* hybridisation (RISH) technique, to detect *Mtb* mRNA in ante- and postmortem human TB tissues and developed a dual ZN/immunohistochemistry staining approach to identify AFB and bacilli producing antigen 85B (Ag85B).

**Findings:**

We identified *Mtb* mRNA within intact and disintegrating bacilli as well as extrabacillary mRNA. *Mtb* mRNA was distributed zonally within necrotic and non-necrotic granulomas. We also found *Mtb* mRNA within, and adjacent to, necrotic granulomas in ZN-negative lung tissue and in Ag85B-positive bronchiolar epithelium. Intriguingly, we observed accumulation of *Mtb* mRNA and Ag85B in the cytoplasm of host cells. Notably, many AFB were negative for Ag85B staining. *Mtb* mRNA was observed in ZN-negative antemortem lymph node biopsies.

**Interpretation:**

RNAscope and dual ZN/immunohistochemistry staining are well-suited for identifying subsets of intact *Mtb* and/or bacillary remnants in human tissue. RNAscope can identify *Mtb* mRNA in ZN-negative tissues from patients with TB and may have diagnostic potential in complex TB cases.

**Funding:**

Wellcome Leap Delta Tissue Program, Wellcome Strategic Core Award, the 10.13039/100000002National Institutes of Health (NIH, USA), the Mary Heersink Institute for Global Health at UAB, the UAB Heersink School of Medicine.

**Research in context**

**Evidence before this study**

The scarcity of tools for consistent detection of intact *Mtb* bacilli, bacillary remnants, or secreted antigens within human tissue hampers investigation into *Mtb* physiology *in vivo* and the spatial distribution of bacilli within host cells and TB lesions. For diagnostic and research purposes, identification of *Mtb* in sputum, needle biopsies, resected specimens, or postmortem tissue relies on microscopic observation of Ziehl-Neelsen (ZN)-stained, acid-fast *Mtb* bacilli (AFB). However, acid fastness in *Mtb* is highly variable, and ZN staining is not *Mtb*-specific.

**Added value of this study**

We used RNAscope, an RNA *in situ* hybridisation (RISH) technique, to detect *Mtb* mRNA within intact and disintegrating bacilli in ZN-positive and -negative human TB tissue. Employing RNAscope and dual ZN/immunohistochemistry (IHC) staining, we found that *Mtb* mRNA and secreted antigens accumulate within cells in histologically normal and diseased airways, including bronchiolar epithelial cells. This finding enhances our understanding of the historical imprints left by *Mtb in vivo* and sheds light on clinically significant mechanisms of immune subversion. Further, the identification of two phenotypically distinct *Mtb* populations based on differential Ag85B expression may provide unique insight into efficacy of anti-TB drugs, transmission dynamics, and immune mechanisms involved in bacillary clearance. Importantly, we identified *Mtb* mRNA in a ZN-negative antemortem biopsy from a patient that was initially diagnosed with histoplasmosis but was diagnosed with TB following autopsy.

**Implications of all the available evidence**

Our findings have important implications for the study of *Mtb* physiology *in vivo* and for TB diagnosis in complex cases where biopsy material is ZN-negative. Firstly, despite assumptions that *Mtb* mRNA instability would limit its use as a marker of infection, we observed intra- and extracellular RNAscope signal patterns in antemortem and postmortem human TB tissues. Secondly, detection of subsets of *Mtb* mRNAs in tuberculous tissue may facilitate assessment of *Mtb* viability, alternate physiological states, or treatment efficacy and is expected to contribute to a more nuanced understanding of the temporal aspects of *Mtb* infection. Thirdly, our observation that discrete bacilli in close proximity exhibit differential Ag85B expression suggests that host environmental signals are unlikely to influence expression of this antigen, and that Ag85B immunostaining alone would underestimate bacillary burden. Finally, the diagnostic capacity inherent in the detection of *Mtb* mRNA in ZN-negative human tissue implies that RNAscope holds promise as a diagnostic tool across a diverse spectrum of human pathogens.

## Introduction

Tuberculosis (TB) continues to be a threat to global health, with significant morbidity and mortality. Accordingly, the capacity to detect *Mtb* bacilli accurately and consistently is critical for definitive TB diagnosis and research efforts. Culturing of clinical isolates remains the gold standard for TB diagnosis but takes weeks to complete. Alternatively, interferon gamma release assays are widely used to diagnose active or latent TB using whole blood.^1^, ^2^, ^3^ More rapid diagnostic approaches that rely on PCR amplification of specific *Mtb* genomic sequences are highly sensitive and are well-suited for detection of *Mtb* in paucibacillary specimens.^4^, ^5^, ^6^, ^7^, ^8^ However, despite the important diagnostic utility of these and other molecular approaches, few methods exist that allow consistent spatial detection of *Mtb* bacilli, bacillary remnants, or secreted antigens within human tissue, which limits investigation of *Mtb* physiology and bacillary distribution.

Detection of *Mtb* bacilli in tissue samples has traditionally relied on microscopic observation of acid-fast bacilli (AFB) following Ziehl-Neelsen (ZN) histochemical staining. However, since *Mtb* can be cultured from ZN-negative tissue specimens, ZN staining can produce false negatives, delaying therapeutic intervention.^9^^,^^10^ Further, ZN staining provides no insight into the physiological state of *Mtb, i.e*., whether the bacillus is alive or dead. Importantly, studies in the 1940s showed that single *Mtb* colonies are stratified into three layers containing non-acid-fast, weakly acid-fast, and strongly acid-fast bacilli.^11^ Also, in the 1950s, Canetti reported the presence of “ghost” bacilli in the caseum characterized as translucent, disintegrating cells with variable acid-fastness, which became scarcer as the age of necrosis increased.^12^ Unfortunately, the implications of this important biological property, *i.e*., acid-fastness, have been underappreciated. Since *Mtb* can transition from ZN-positive to ZN-negative *in vivo* and in response to drug therapy,^13^ there is a strong unmet need for improved detection of *Mtb* in human specimens.

One approach used to bypass the limitations of ZN and Auramine O staining is detection of *Mtb* protein antigens in clinical samples by immunohistochemistry (IHC).^14^, ^15^, ^16^, ^17^ *Mtb* has highly regulated secretion systems that contribute to its virulence and secreted antigens play diverse roles in promoting TB disease.^18^ *Mtb* in culture has been shown to secrete over one thousand protein antigens.^19^ These include abundant, well-studied CFP-10,^20^ ESAT-6,^21^^,^^22^ MPT64^23^^,^^24^ and the antigen 85 complex comprised of Ag85A, B and C proteins.^25^^,^^26^ However, while these antigens are expressed within *Mtb*-infected host cells, little is known about the distribution of *Mtb* antigens in human TB lesions.^16^^,^^27^, ^28^, ^29^, ^30^ Thus, despite the utility of antibodies for detecting *Mtb* surface or secreted antigens,^24^^,^^31^, ^32^, ^33^, ^34^ diagnosis of TB based on IHC alone is problematic due to insufficient specificity and challenging standardisation.

Strategies based on *in situ* hybridisation (ISH)^35^ using oligonucleotide probes have been used to detect *Mtb* DNA^36^, ^37^, ^38^^,^ rRNA^39^, ^40^, ^41^, ^42^ or mRNA^43^ in clinical specimens. However, studies comparing RNA *in situ* hybridisation (RISH) methods with routine histochemistry and/or immunohistochemistry (IHC) for the spatial identification of *Mtb* and *Mtb* mRNA are lacking, especially in human paucibacillary and abacillary tissues. A novel RISH platform, referred to as RNAscope, was recently developed.^44^ RNAscope uses an innovative probe design to increase specificity and the signal-to-noise ratio to allow visualisation of single mRNA molecules (as puncta) within cells in formalin-fixed paraffin-embedded (FFPE) tissue.^44^, ^45^, ^46^, ^47^, ^48^ Since the quantity of a specific *Mtb* mRNA molecule can exceed that of its corresponding gene by orders of magnitude, RNAscope allows assessment of the spatial and microenvironmental distribution of mRNA *in vivo*, thereby providing further insight into mycobacterial physiology and heterogeneity.

Therefore, the goal of this study was to determine the spatial distribution of *Mtb* bacilli and/or bacillary remnants, including mRNA and secreted antigens, within distinct microenvironments in human TB lesions. To this end, we adapted RNAscope to detect *Mtb* in ZN-positive human pulmonary and extrapulmonary TB tissue and in ZN-negative ante- and postmortem tissue with proven active TB. Further, we employed IHC to detect *Mtb* antigens to investigate whether *Mtb* bacilli in human tissue exist as a homogenous population regarding antigen production.

## Methods

### Ethics and human subjects

This study was approved by the University of KwaZulu-Natal Biomedical Research Ethics Committee (BREC; class approval study number BCA 535/16, and BE019/13) and the University of Alabama at Birmingham (UAB) Institutional Review Board (IRB; study numbers IRB-300008174 and IRB-300008174-2). Consent for research use of autopsy material is included in the UAB authorization for autopsy consent form signed by the decedent's next of kin.

*Mtb*-infected lung tissues were obtained from three HIV-positive patients with TB undergoing lung resection for removal of irreversibly damaged lobes or lungs (bronchiectasis and/or cavitary lung disease) at King DinuZulu Hospital Complex, a tertiary center for patients with TB in Durban, South Africa. Written informed consent was obtained from all participants. All patients undergoing lung resection for TB had completed a full 6- to 9-month course of anti-TB treatment or up to two years of treatment for drug-resistant TB. Patients were assessed for the extent of pulmonary disease (cavitation and/or bronchiectasis) via high-resolution computed tomography (HRCT). The fitness of each patient to withstand a thoracotomy and lung resection was determined by using the Karnofsky score, 6-min-walk test, spirometry, and arterial blood gas measurement. Assessment of patients with massive hemoptysis included their general condition, effort tolerance before hemoptysis, arterial blood gas measurement, serum albumin concentration, and HRCT imaging of the chest. On gross assessment, all pneumonectomies or lobectomies were bronchiectatic, hemorrhagic, variably fibrotic, and atelectatic and contained visible tubercles.

*Mtb*-infected testicular tissue with features of tuberculous epididymo-orchitis was obtained from a HIV-positive patient with TB following resection. Granulomas showed caseous necrosis and numerous AFB were identified.

Lung tissue from an HIV-negative neonate was obtained postmortem from each lobe of the left and right lung. No infectious pathogens, granulomata, or neoplastic infiltrates were observed.

Antemortem tissues from CT-guided needle biopsies (inguinal lymph node, retroperitoneal lymph node, and bone marrow) and postmortem tissues (lung, periaortic lymph node, liver, and bone marrow) were obtained from an HIV-negative patient undergoing treatment at the University of Alabama at Birmingham Hospital in Birmingham, Alabama. Given the small number of surgically resected and postmortem tissue specimens, sex and gender were not taken into consideration in the study design. Sex, stated as male and female, was self-reported. See Supplemental Table S1 for additional details regarding human subjects and tissues.

### Histology

Human tissue specimens processed at the Africa Health Research Institute (AHRI) were aseptically removed and fixed in 10% neutral buffered formalin (Sigma–Aldrich, cat # HT501850). Specimens were processed in a Sakura Tissue-Tek VIP6 vacuum filtration tissue processor using a xylene-free protocol and embedded in paraffin using a Thermo Fisher Histostar embedding station. For H&E staining, the formalin-fixed, paraffin-embedded (FFPE) tissue blocks were cut into 4 μm sections, mounted on Superfrost Plus charged slides (Thermo Fisher cat # 22-037-246), heated at 56 °C for 15 min, deparaffinised through 2 changes of xylene and rehydrated through descending grades of alcohol to water. H&E staining was performed by placing slides in hematoxylin for 5 min, washing in tap water for 2 min, bluing in lithium carbonate for 1 min, rinsing in tap water for 2 min and counter staining with eosin for 5 min before a final rinse in tap water for 2 min. Slides were dehydrated in ascending grades of alcohol, cleared in xylene, and the coverslip was mounted using DPX (Distyrene, Plasticizer, and Xylene, Sigma–Aldrich, cat # 06522). For ZN staining, FFPE tissue blocks were cut into 2 μm sections, mounted on charged slides, and heated at 56 °C for 15 min. Mounted tissue sections were dewaxed in xylene followed by rinsing in 100% ethanol and 1 change of 95% ethanol. Slides were incubated with heated carbol fuchsin (MediaMage, cat #M00385) for 15 min and then washed in running tap water. Three percent acid alcohol was applied to the slide for 30 s to decolorize or until sections appeared clear. Slides were then washed in running tap water for 2 min, then counterstained with methylene blue. Slides were rinsed under running water, dehydrated, and the coverslip was mounted using DPX.

Human tissue specimens processed at UAB were fixed in 10% neutral buffered formalin and embedded in paraffin. H&E, ZN, and Grocott-Gomori Methenamine Silver (GMS) staining were performed in the UAB Anatomic Pathology Laboratory according to standard clinical laboratory protocols. Prior to staining, formalin-fixed, paraffin-embedded (FFPE) tissue blocks were cut into 4 μm sections and mounted on glass slides. H&E staining was performed using a Leica Autostainer XL automated slide stainer that performs deparaffinisation, dehydration, staining, dehydration, and clearing steps using VENTANA reagents (Roche Diagnostics). Following deparaffinisation, ZN staining was performed essentially as detailed above, except slides were incubated with carbol fuchsin for 1 h. Following deparaffinisation, GMS staining was performed by hydrating sections in distilled water followed by exposure to 5% chromic acid for 30 min at RT. After rinsing in tap water, slides were placed in a 2% sodium bisulfite solution for 5 min and rinsed with distilled water. Slides were then transferred to methenamine-silver nitrate solution (a combination of 20 mL of 5% silver nitrate solution, 400 mL of a 3% methenamine solution, and 2 mL of 3% sodium borate) for 30 min at 56 °C, or until full color development. Slides were then placed in a 0.5% gold chloride solution for 5 min, rinsed with distilled water, and transferred into 5% sodium thiosulfate for 5 min. After rinsing with distilled water, slides were counterstained with 0.1% light green solution for 3 min, rinsed, and dehydrated. Coverslips were mounted with Richard-Allan Scientific Cytoseal XYL (Thermo Fisher cat # 22-050-262).

### Immunohistochemistry

FFPE tissues were cut into 2 μm thick sections, mounted on charged slides, and heated at 56 °C for 15 min on a hotplate. Mounted sections were dewaxed in two changes of xylene followed by rinsing in 2 changes of 100% ethanol and 1 change of 95% ethanol. Slides were then rinsed in tap water for 2 min followed by antigen retrieval via Heat Induced Epitope Retrieval (HIER) in trisodium citrate (pH 6.0) for 30 min. Slides were cooled for 15 min and rinsed in tap water for 2 min. Endogenous peroxide activity was blocked using 3% hydrogen peroxide (Leica Biosystems Novolink Polymer Detection Systems, cat # RE7157) for 10 min at room temperature (RT). Slides were then rinsed in phosphate-buffered saline containing 0.1% Tween 20 (PBST) and blocked with protein block (Leica Novolink Systems, cat # RE7158) for 5 min at RT. Sections were incubated with unconjugated primary antibody directed against *Mtb* antigen 85B (Ag85B; Abcam cat # ab43019, RRID:AB_776575, 1:500), *Mtb* Early Secreted Antigenic Target 6 (ESAT-6; Abcam cat # ab26246, RRID:AB_449032, 1:500) or *Mtb* Uncharacterised Surface Protein (USP; Lifespan Bioscience cat # LS-C683286, 1:500) followed by rinsing in PBST and incubated with enzyme-linked polymer (Leica Novolink Systems, cat # RE7161) for 30 min at RT. Slides were then rinsed and stained with 3,3′-Diaminobenzidine (DAB) chromogen (Leica Novolink Systems, cat # RE7162) for 5 min, rinsed under running water and counterstained with hematoxylin for 2 min. Slides were rinsed in tap water, blued in 3% ammoniated water for 30 s, rinsed in tap water, dehydrated in ascending grades of alcohol, cleared in xylene, and the coverslip was mounted using DPX.

### RNAscope

The RNAscope 2.5 High Definition (HD)-Red assay kit (Advanced Cell Diagnostics [ACD] cat # 322350) employed here uses a red chromogen to visualise target mRNA. All RNAscope probe sets were purchased from ACD. To ensure probe set specificity, a design algorithm was used to examine each probe for potential cross-reactivity against the host cell transcriptome (for detecting single mRNAs within eukaryotic or prokaryotic cells) or against transcriptomes of related organisms (for identifying specific organisms). We used a positive control RNAscope probe set comprised of 16 probe pairs directed against bp 139–989 of human peptidylprolyl isomerase B (PPIB) mRNA (ACD cat # 313901). The negative control probe set consists of 10 probe pairs directed against bp 414–862 of *Bacillus subtilis* dihydrodipicolinate reductase (*dapB*) mRNA (ACD cat # 310043). The *Mtb*-specific probe set (ACD cat # 552911) consists of 120 probe pairs directed against mRNAs from six genes (20 probe pairs per gene): secreted l-alanine dehydrogenase (*ald*), catalase-peroxidase-peroxynitritase T (*katG*), trehalose-6-phosphate phosphatase (*otsB1*), resuscitation-promoting factor A (*rpfA*), resuscitation-promoting factor B (*rpfB*), and ATP-binding protein (ABC transporter) (*irtB*).

RNAscope 2.5 High Definition (HD)-Red assays were performed essentially as recommended by the manufacturer, with modifications as stated below. Briefly, FFPE human lung, testicle, and lymph node tissue blocks were cut into 4 μm sections and mounted on Superfrost Plus charged slides. Control RNAscope slides (ACD cat # 310045) premounted with thin sections of a FFPE HeLa cell pellet were also used. Slides were heated at 60 °C for 1 h on a hotplate and deparaffinised at RT in two changes of xylene followed by two changes of 100% ethanol to remove the xylene. Tissue sections were pretreated with hydrogen peroxide (ACD cat # 322381) for 10 min and rinsed with distilled water. Target retrieval was performed by incubating the tissue sections in 1x RNAscope target retrieval reagent (ACD cat # 322000) at 100 °C for 30 min (lung, testicular and lymph node tissue) or 15 min (control HeLa cell pellet). Sections were immediately rinsed in distilled water, then in 100% ethanol, and air died. A hydrophobic barrier was created around the tissue section using a ImmEdge hydrophobic barrier pen (ACD cat # 310018). All tissues underwent protease treatment by applying RNAscope Protease Plus solution (ACD cat # 322381) onto each tissue section at 40 °C for 30 min (lung, testicular and lymph node tissue) or 20 min (control HeLa cell pellet) in a HybEZ oven (ACD cat# 321711) to permeabilise cells and increase probe accessibility. Prior to addition of RNAscope probe sets, the solubilized probes were heated at 40 °C for 10 min and cooled to RT. RNAscope probes were dropped onto the tissue sections which were placed in a HybEZ oven (ACD cat # 321711) for 2 h at 40 °C. Slides were then washed in 2 changes of fresh 1x RNAscope wash buffer (ACD cat # 310091) for 2 min at RT. A series of six signal amplification steps followed, each comprised of dropping the amplifier reagent on the section followed by incubation for 15 or 30 min at either RT or 40 °C, with each step followed by 2 rinses in 1x wash buffer according to the manufacturer's protocol. Following amplification, 120 μl of red chromogen solution (a 1:60 ratio of RED-B and RED-A) was dropped onto the sections and left at RT for 10 min. Excess chromogen was then tilted off the slides followed by rinsing in 1 change of distilled water and 1 change of tap water. Tissue sections were then counterstained with a 50% hematoxylin and water solution at RT for 2 min followed by 3 dips in 0.02% ammonia water to visualise cell nuclei. Slides were then washed in 2 changes of tap water followed by drying at 60 °C for 15 min. Coverslips were mounted using RNAscope VectaMount medium (ACD cat # 321584) which is compatible with the red chromogen. Imaging was performed on a slide scanner as stated below.

### Microscopy and imaging

Resected lung specimens (tuberculous and neonatal), testicle, and antemortem inguinal lymph node needle biopsy specimens examined by H&E, ZN, IHC (*Mtb* Ag85B, ESAT-6, USP) or RNAScope were imaged at AHRI using a Hamamatsu NDP slide scanner (NanoZoomer RS2, Model C10730-12) using its NDP.View2 viewing software. Antemortem inguinal and retroperitoneal lymph node specimens and postmortem lung, liver, bone marrow and periaortic lymph node specimens examined by H&E, ZN, and GMS staining were imaged at UAB using an Olympus BX50 microscope with a DP23 digital microscope camera with Olympus cellSens Entry 3.2 image acquisition software (Build 23706). Automatic exposure settings were used after manual white balance. Image resolution was set at “Full 3088 x 2076”.

### HALO® analysis

Whole-slide image analysis was performed using Halogen-Assisted Light Optimisation (HALO) v3.6.4134 (Indica Labs, Corrales, NM). HALO uses image analysis algorithms to automate the analysis and quantitation of RNAscope *Mtb* mRNA puncta in TB tissue specimens. HALO was used to calculate the number of mRNA puncta, measure puncta intensity, and assess spatial distribution. Regions of interest (ROIs) were drawn using the annotation tools provided in the HALO platform. The ISH module v4.2.3 (Indica Labs, Corrales, NM) was used to detect RNAscope signal puncta. For visualisation, the Area Quantification module v2.4 was used. For both modules, the magnification was set to “1” with the parameters described in Supplementary Table S2.

### Statistics

Results are shown as Mean ± SD. All graphs were plotted using GraphPad Prism v6.04 (GraphPad Software Inc., USA) and the statistical significance was calculated by applying Student's t-test or One-way ANOVA. Differences were considered statistically significant at P values less than 0.05. Exact P values are included in the data plots for statistical evaluation.

### Role of funders

The funders of this work played no part in the experimental design, data collection, data analysis and/or interpretation, or creation of this manuscript.

## Results

### RNAscope cellular and tissue controls

**Fig. 1:**
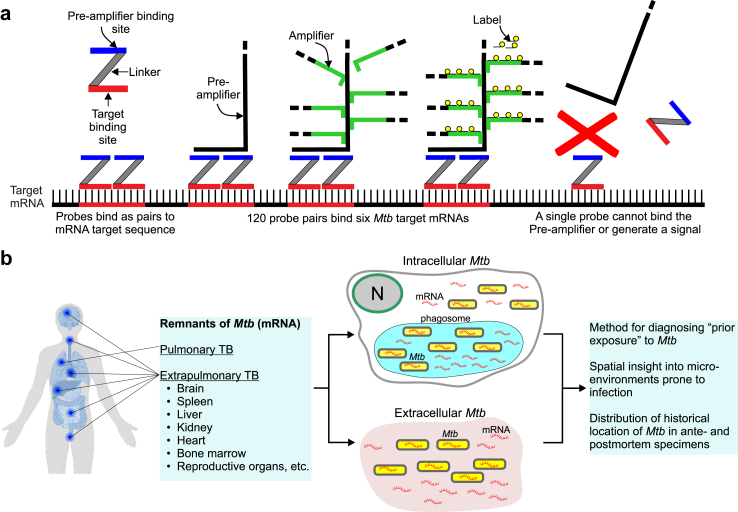
**RNAscope and its application for detecting *Mtb* in human tissues**. (**a**) Schematic of the RNAscope *in situ* hybridisation platform for mRNA detection. Following tissue fixation and permeabilisation, up to 20 “Z” oligonucleotide probe pairs hybridise with multiple target sequences (red lines) within a single mRNA across a ∼1000-bp region. For detection of *Mtb* in human tissue, 120 probe pairs (20 pairs per mRNA) bind complimentary sequences in six *Mtb* mRNAs. Subsequent steps include binding of a pre-amplifier to mRNA-bound “Z” only to probe pairs, addition of amplifiers molecules, and binding of chromogen label (fluorescent labels can also be used) for detection via microscopy where an individual mRNA molecule appears as a single dot or punctum. The binding of a single “Z” probe to mRNA does not result in pre-amplifier binding or a signal. (**b**) Diagram illustrating the potential of RNAscope to identify and track *Mtb* mRNA in resected, antemortem, or postmortem specimens in pulmonary or extrapulmonary tissue. Schematic in **(a)** adapted from Anderson, *et al*.^49^

RNAscope is a highly specific and sensitive RISH technique that combines multiple paired “Z” oligonucleotide probes with signal amplification steps to allow visual detection of single mRNA molecules in tissue samples as colored dots, or puncta, while preserving tissue architecture^44^ (Fig. 1a). Since a single mRNA molecule can be bound by one probe pair resulting in a faint punctum, or by several probe pairs resulting in an intensely-colored punctum, it is the number of puncta, not the color intensity of individual puncta, that reflects the abundance of target mRNAs. We posited that RNAscope could detect mRNA in bacilli, infected host cells, and antemortem and/or postmortem human specimens (Fig. 1b). We first validated RNAscope assays by exposing thin sections of a HeLa cell pellet to a positive control probe set directed against human peptidylprolyl isomerase B (*PPIB*) mRNA (Supplemental Figure S1a) or a negative control probe set specific for *Bacillus subtilis* dihydrodipicolinate reductase (*dapB*) mRNA (Supplemental Figure S1b) resulting in robust signals and no signal, respectively. The *Mtb*-specific RNAscope probe set (120 probe pairs) targets six mRNAs to provide specificity and sensitivity. We tested the *Mtb* probe set against human neonatal lung tissue, which showed no signal as expected (Supplemental Figure S1c). Lastly, to verify the integrity of human specimens for RNAscope analysis, we applied the *PPIB* positive control probe set to testicular tissue from a patient with TB which showed robust signals (Supplemental Figure S2). Overall, these control assays demonstrate that RNAscope is compatible with the tissue processing protocols for our human specimens.

### RNAscope detects intact and disintegrated *Mtb* bacilli, and mRNA in human tissue

**Fig. 2:**
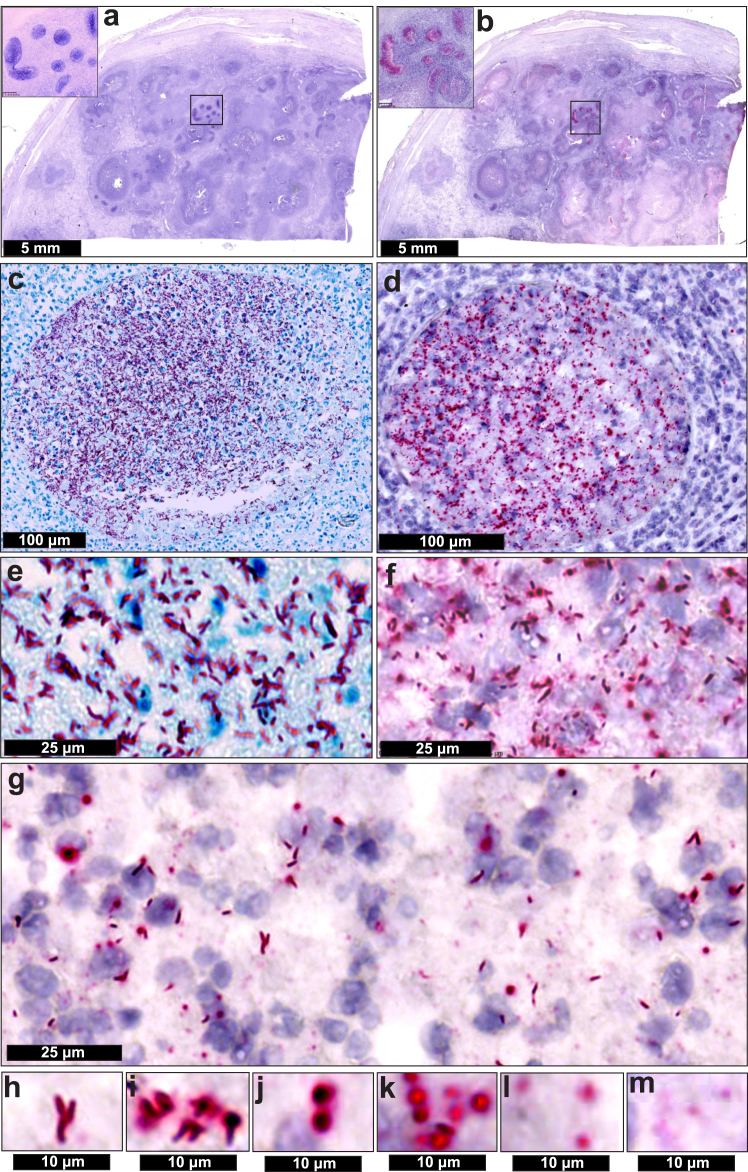
**RNAscope detects intact and disintegrated *Mtb* bacilli and mRNA in human tissue**. (**a**) low power image of a human testicular specimen containing ZN-positive AFB. Inset–note positive ZN staining within the seminiferous tubules. (**b**) low power image of a consecutive section of the testicular specimen in (**a**) revealing RNAscope *Mtb* mRNA signals, which are abundant within the seminiferous tubules. Medium power image showing ZN-positive AFB (**c**) or RNAscope *Mtb* signals (**d**) within seminiferous tubules. High power image revealing ZN-positive AFB (**e**) and RNAscope *Mtb* mRNA signals (**f**). (**g**) High power image showing a spectrum of RNAscope *Mtb* mRNA signals within a single field **(h**–**m).** High power images of RNAscope signals from (**h**) intact bacilli, (**i, j**) partially intact bacilli, (**k, l**), disintegrated bacilli appearing as large, diffuse puncta (**m**) small puncta representing single extrabacillary *Mtb* mRNAs.

To compare the ability of RNAscope and ZN staining to detect *Mtb* bacilli *in situ*, we examined seminiferous tubules containing AFB obtained from a patient with active TB. Since the structurally complex *Mtb* cell wall is considered a barrier^13^^,^^50^ to oligonucleotide probes, conditions were optimised to allow *Mtb* RNAscope probes to enter intact bacilli. RNAscope identified *Mtb* bacillary rods inside and outside the seminiferous tubules (Fig. 2b, d, f) that are visually similar to ZN-stained bacillary rods (Fig. 2a, c, e). RNAscope yielded a spectrum of signal shapes: bacilli with halos, bacillus-sized puncta (consistent with the diameter of a vertical bacillus within the cut plane), diffused halos, and smaller puncta (Fig. 2f, g, h–m), which were also observed within host cells (Fig. 2g).

In summary, improved permeabilisation permits RNAscope probes to detect *Mtb* bacillary rods. Secondly, a halo surrounding bacilli suggests mRNA leakage, indicating cellular disintegration that supports Canetti's findings of bacillary disintegration and loss of acid-fastness *in vivo*.^12^ Thirdly, our findings show that *Mtb* mRNA is stable enough for detection with RNAscope in numerous microenvironments. These findings provide new biological insight into bacillary morphology *in vivo* and cell death, which cannot be obtained through ZN staining.

### RNAscope detects *Mtb* mRNA in ZN-negative human TB lung tissue

**Fig. 3:**
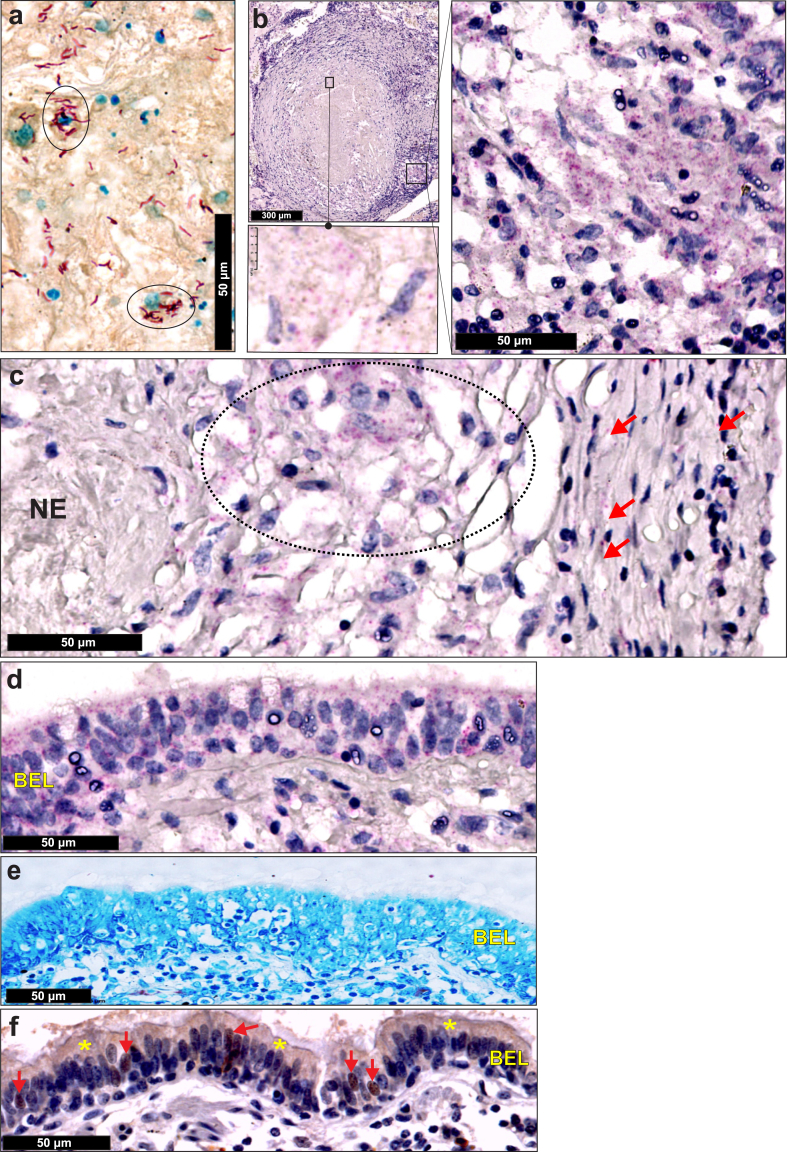
**RNAscope detects *Mtb* mRNA in human TB lung tissue.** Lung tissue from a patient with active TB. (**a**) ZN-positive tissue section revealing numerous intracellular (circled) and extracellular AFB. (**b**) ZN-negative lung tissue specimen from the patient in (**a**) containing a necrotic granuloma with RNAscope *Mtb* mRNA puncta; boxed regions correspond to high power images showing RNAscope puncta. (**c**) High power image revealing RNAscope *Mtb* mRNA puncta within a necrotic area (NE), granulomatous area (oval) and granulomatous inflammatory layer (red arrows). (**d**) High power image of numerous RNAscope *Mtb* mRNA puncta within the bronchiolar epithelial layer (BEL). (**e**) ZN staining showing the absence of AFB in the BEL. (**f**) High power image of Ag85B-positive staining within the cytoplasm (yellow asterisks) and nuclei (red arrows) of bronchiolar epithelial cells.

We next examined the utility of RNAscope for identifying *Mtb* or remnants of infection, *i.e*., mRNA, in ZN-negative human TB tissue. ZN staining of formalin-fixed, paraffin-embedded (FFPE) lung tissue from a patient with active TB showed numerous extracellular and intracellular bacilli (Fig. 3a). However, a different FFPE lung tissue specimen from the same patient was ZN-negative. This is unsurprising as most human pulmonary TB granulomas contain few, if any, ZN-positive *Mtb*, which is consistent with historical studies.^12^ In contrast, RNAscope analysis of this ZN-negative tissue revealed numerous RNAscope signal puncta within necrotic granulomas (Fig. 3b and c). RNAscope puncta were also abundant in bronchiolar epithelial cells (Fig. 3d), which were ZN-negative (Fig. 3e) and antigen 85B (Ag85B)-positive (Fig. 3f).

These data demonstrate that *Mtb* mRNA is abundantly present in ZN-negative lung tissue sections of a confirmed case of pulmonary TB and that *Mtb* mRNA is sufficiently stable for detection by RNAscope. RNAscope and Ag85B immunostaining suggest the bronchiolar epithelial layer as an overlooked entry portal for *Mtb* infection.

### *Mtb* mRNA and secreted antigens accumulate in the cytoplasm of host cells

**Fig. 4:**
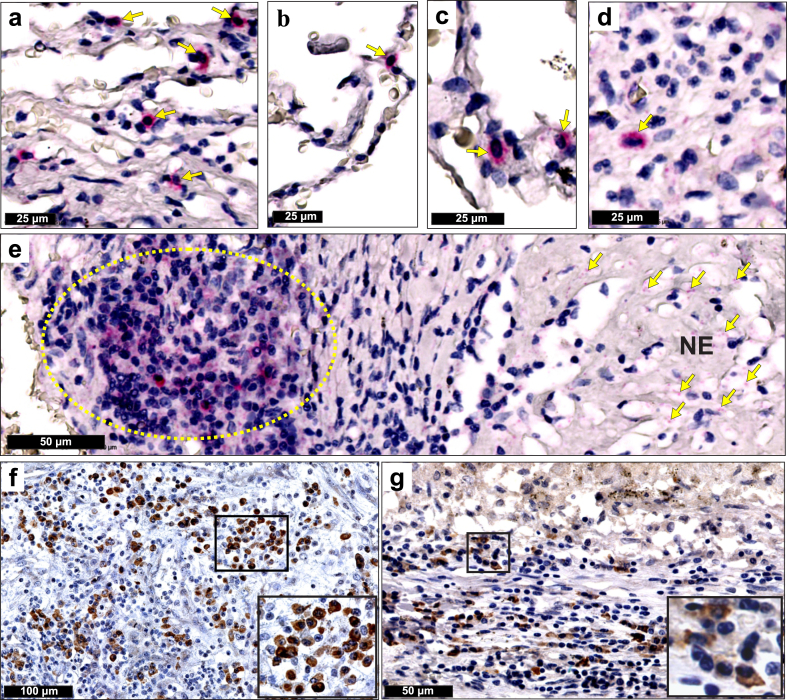
**Accumulation of *Mtb* mRNA and secreted antigens in cells from pulmonary and extrapulmonary tissue**. **(a–e, g)** The same ZN-negative lung tissue specimen analysed in Fig. 3b–f. (**a**–**d**) High power images showing prominent RNAscope *Mtb* mRNA signals within alveolar epithelial cells (yellow arrows). (**e**) High power image of RNAscope signals within lymphoid aggregates of necrotic granulomas (area in yellow oval). **(f)** Medium power image of Ag85B-positive staining in the cytoplasm of macrophages in the ZN-positive extrapulmonary (testicular) human tissue specimen shown in Fig. 2. (**g**) Medium power image of Ag85B-positive staining in the cytoplasm of alveolar macrophages and epithelioid histiocytes within pulmonary human TB specimens. (**f**, **g**) Insets show higher power images of the cytoplasmic localisation of Ag85B staining.

In a study of individuals who died from causes other than TB, *Mtb* DNA was detected in alveolar and interstitial macrophages, type II pneumocytes, endothelial cells, and fibroblasts via *in situ* PCR analysis.^51^ Even though AFB were not shown, these findings are in accord with early reports^52^, ^53^, ^54^, ^55^ that *Mtb* can persist in lung tissue without histological evidence of TB lesions. Consistent with those studies, we identified accumulated *Mtb* mRNA in the cytoplasm of several alveolar epithelial cells (Fig. 4a–d) in a signal pattern distinct from the intracellular puncta that represent single *Mtb* mRNA molecules. We refer to such cells as host-cell accumulated *Mtb* RNA (HAMR) cells. *Mtb* mRNA is present in HAMR cells within adjacent regions (Fig. 4b and c), consolidated alveolar areas (Fig. 4d), and within lymphocytic aggregates at the periphery of necrotic granulomas (Fig. 4e). The functional significance of *Mtb* mRNA within HAMR cells is unknown; however, these mRNAs may act as pathogen-associated molecular patterns (PAMPs) as has been shown for mRNAs in conventional innate immune cells^56^, ^57^, ^58^, ^59^, ^60^ to modulate immunity.

Secreted *Mtb* antigens have been identified via IHC staining in *Mtb*-infected human lung tissue.^24^^,^^31^, ^32^, ^33^, ^34^ Similarly, we used IHC to detect *Mtb* secreted antigen 85B (Ag85B) in the same pulmonary and testicular specimens used for RNAscope analysis (Fig. 2, Fig. 3). We observed Ag85B accumulation in numerous cell types (Fig. 4f) including alveolar macrophages and epithelioid histiocytes (Fig. 4g). In summary, our data demonstrate that cells from histologically normal and diseased airways accumulate *Mtb* mRNA and secreted antigens.

### Spatial distribution of *Mtb* RNA in host microenvironments

**Fig. 5:**
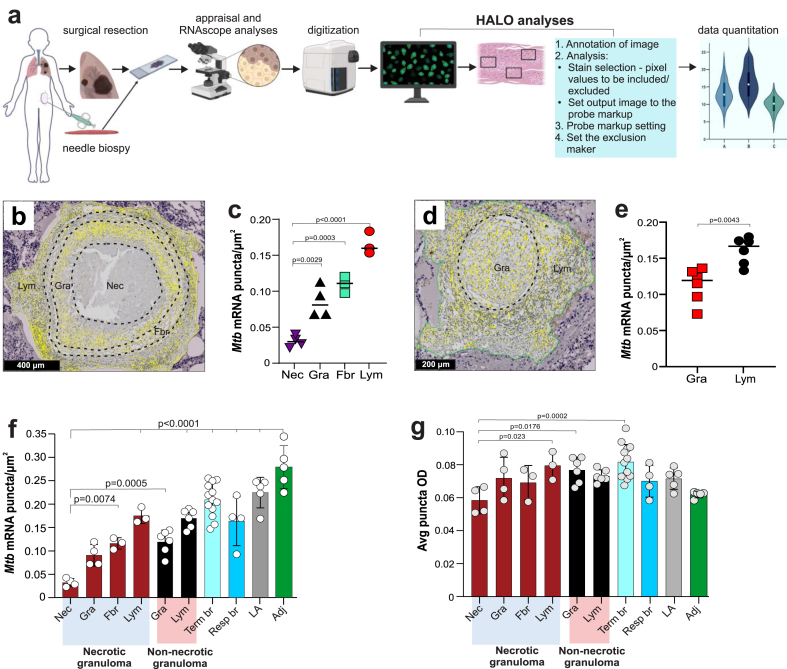
**Quantitation of RNAscope signals to inform the biology of TB lesions**. (**a**) Flow diagram depicting sample acquisition, RNAscope analysis, digitisation, and quantitation of RNAscope *Mtb* mRNA puncta using HALO**®** analysis software. (**b**–**g**) The same ZN-negative lung tissue specimen analysed in Fig. 3, Fig. 4. (**b**) Representative zonation of a single necrotic granuloma and (**c**) number of RNAscope *Mtb* mRNA puncta per square micron in each lesion zone. (**d**) Representative zonation of a single non-necrotic granuloma and (**e**) number of RNAscope *Mtb* mRNA puncta per square micron in each lesion zone. Yellow puncta (**b** and **d**) are false-color RNAscope *Mtb* mRNA signals. (**f**) Number of RNAscope *Mtb* mRNA puncta per square micron and (**g**) average puncta optical density (OD) in TB lesions and tissue. Zones include necrotic (Nec), granulomatous inflammatory (Gra), fibrotic (Fbr), lymphocytic (Lym), terminal bronchiole (Term br), respiratory bronchiole (Resp br), lymphocytic aggregates (LA) and tissue adjacent to diseased areas (Adj). In (**c**, **e**, **f, and g**), each data point represents the number of RNAscope signal puncta per square micron (**c**, **e**, and **f**) or average puncta OD (**g**) in an individual zone within a granuloma or other lung feature. In (**c, f, and g**), four necrotic granulomas were examined that exhibited Nec and Gra zones (n = 4) and Fbr and Lym zones (n = 3). In (**e, f, and g**), six non-necrotic granulomas were examined that contained Gra and Lym zones (n = 6). (**f**, **g**) Term br; n = 13, Resp br; n = 4, LA; n = 5, Adj; n = 5. Data in (**c**, **e**, **f** and **g**) represent the mean ± SD. Data were analysed using one-way ANOVA and Bonferroni's multiple comparison test (**c**, **f,** and **g**) or by unpaired Mann–Whitney test (**e**). (**c**, **f, and g**) all comparisons are with respect to the Nec zone.

Our findings show that RNAscope can identify *Mtb* mRNA to reveal prior history of infection, which is not possible with ZN or Auramine O staining. Since each RNAscope punctum represents one *Mtb* mRNA molecule, we used the HALO® image analysis platform to count *Mtb* mRNA puncta, expressed as the number of *Mtb* mRNA puncta per square micron, in necrotic and non-necrotic granulomas, terminal and pulmonary bronchi, lymphocytic aggregates, and adjacent tissue from resected TB lung tissue (Fig. 5a). To avoid potentially confounding variables such age, sex, tissue pathology bias, and experimental variability, we selected a single ZN-negative TB tissue specimen containing the aforementioned microenvironments.

We reasoned that quantifying *Mtb* mRNA puncta within each of the four distinct regions of the necrotic granuloma (necrotic, granulomatous inflammatory, fibrotic, and lymphocytic; Fig. 5b) could help inform the pathophysiology of granuloma formation. We found that the necrotic zone contains the fewest *Mtb* mRNA puncta per square micron, followed by increases in the number of puncta within the granulomatous inflammatory, fibrotic, and lymphocytic zones (Fig. 5c), indicating that *Mtb* mRNA is distributed in accordance with discrete pathophysiological zones that may contribute to granuloma formation. We also classified non-necrotic granulomas (Fig. 5d) into lymphocytic and non-necrotic zones and found that the lymphocytic zone exhibited significantly more *Mtb* mRNA puncta than the non-necrotic zone (Fig. 5e).

We next quantified *Mtb* mRNA puncta in terminal and respiratory bronchioles, adjacent tissue, and lymphocytic aggregates distant from necrotic or non-necrotic granulomas and compared these values to those from regions within necrotic and non-necrotic lesions. We found that the number of *Mtb* mRNA puncta in the terminal bronchioles, lymphocytic aggregates, and adjacent tissue were the highest of all areas examined (Fig. 5f). Further, the terminal bronchioles exhibited the highest average puncta optical density (OD) (Fig. 5g), suggesting the presence of longer, more intact *Mtb* mRNAs containing more RNAscope probe binding sequences.

In short, the zonal distribution of *Mtb* mRNA within the granuloma offers a new perspective into the evolution of granuloma formation and clinical course of disease that ZN or other bacillary staining methods cannot. Lastly, the spatial distribution of *Mtb* mRNA provides new insight into the previous impact of *Mtb* within human tissue.

### Identification of phenotypically distinct populations of *Mtb in vivo*

Continuously changing microenvironments within the tuberculous lung, the specific bacillary load, and a variable spectrum of TB lesions^11^^,^^12^^,^^47^^,^^55^^,^^61^^,^^62^ strongly suggest that at least two distinct *Mtb* populations, live and dead, must exist *in vivo*. There is currently no method that accurately distinguishes metabolically living from dead *Mtb* bacilli within tissue specimens; however, since MPT64 is secreted during active *Mtb* growth,^63^ the presence of MPT64 in sputum has been suggested as a marker of *Mtb* viability.^24^ Here, we tested the hypothesis that ZN staining combined with IHC for secreted *Mtb* antigens can identify phenotypically diverse *Mtb* populations *in vivo*. We examined testicular tissue whose seminiferous tubules contain numerous bacilli and are bordered by a basement membrane that separates the tubule from the sparsely-infected surrounding tissue, which aids in evaluating the specificity of *Mtb* antigen immunostaining. H&E histology revealed the presence of substantial cellular debris (Supplemental Figure S4a) which overlaps with *Mtb* RNAscope signals (Supplemental Figures S4b, S3b). *Mtb* bacilli colocalise with strong Ag85B, ESAT-6, and Uncharacterised Surface Protein (USP) positivity in sequential tissue sections (Supplemental Figure S4c-e). While these antigens are typically associated with the *Mtb* cell wall, clear delineation of the bacillary rod shape is not always possible. In some cases (*e.g.*, USP, ESAT-6), positivity was seen as scattered, brown patches, whereas some rod-like shapes (Supplemental Figure S4d, e) were observed, consistent with previous studies.^9^^,^^28^^,^^34^^,^^64^ These findings suggest that Ag85B, ESAT-6, and USP staining patterns may not always match the rod-like morphology of *Mtb*.

**Fig. 6:**
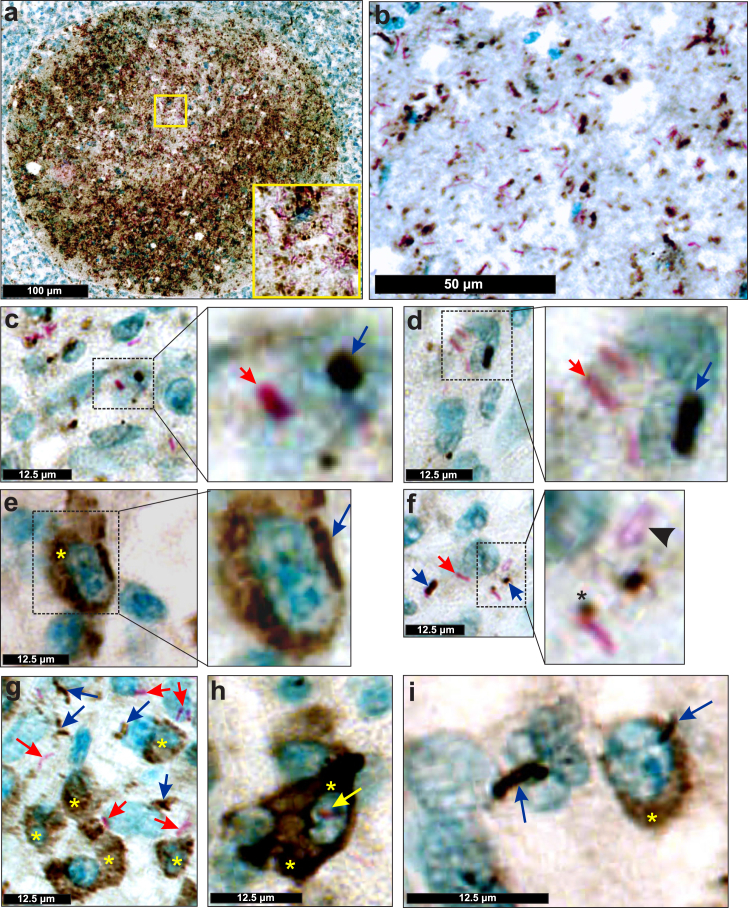
**Phenotypically distinct *Mtb* populations exist within human extrapulmonary TB tissue**. (**a**) Medium power image of a seminiferous tubule with combined ZN staining (pink) and Ag85B IHC staining (brown). Inset; note the presence of Ag85B-positive and -negative bacilli within the seminiferous tubule. (**b**) High power image of ZN/Ag85B staining showing Ag85B-positive bacilli near ZN-positive/Ag85B-negative bacilli. (**c, d**) High power images of ZN/Ag85B dual staining showing Ag85B-positive (blue arrow) and -negative (red arrow) bacilli. **(e)** Ag85B-positive *Mtb* bacillus (blue arrow) and cytoplasm (yellow asterisk). (**f**) translucent, weakly acid-fast “ghost” bacillus (arrowhead) and an AFB with Ag85B positivity at the one pole (asterisk). (**g**) intracellular and extracellular AFB (red arrows) and Ag85B-positive bacilli (blue arrows), with some Ag85B-positive bacilli within Ag85B-positive host cell cytoplasm (yellow asterisks). (**h**) Ag85B-positive host cell cytoplasm (yellow asterisk) with ZN-positive/Ag85B-negative *Mtb* bacillus (yellow arrow). (**i**) Two Ag85-positive *Mtb* bacilli (blue arrows); one is inside an Ag85B-positive host cell (yellow asterisk).

To determine whether distinct microenvironments can contribute to differential antigen production, we examined whether all AFB produce Ag85B. Intriguingly, our dual ZN/Ag85B IHC stain demonstrated that not all AFB produce Ag85B. This was evident by numerous ZN-positive/Ag85B-negative bacilli close to Ag85B-positive bacilli (Fig. 6a–d, Supplemental Figures S5a, b, S6). Ag85-positive and ZN-positive/Ag85B-negative bacilli were observed extracellularly and within host cells that did, or did not, accumulate Ag85B (Fig. 6e–i). Similar to Canetti,^12^ we also identified “ghost” bacilli that are weakly acid fast (Fig. 6f).

Taken together, these data demonstrate that two phenotypically distinct *Mtb* populations exist in human TB tissue: Ag85B-positive and Ag85-negative bacilli. This finding strongly suggests that Ag85B IHC alone may underestimate the number of bacilli. This has important implications for TB diagnosis and pathogenesis since mixed populations of *Mtb* may differently influence diagnosis, pathogenesis, and immunity.

### Using RNAscope and secreted *Mtb* antigens as tools for guiding TB therapy

**Fig. 7:**
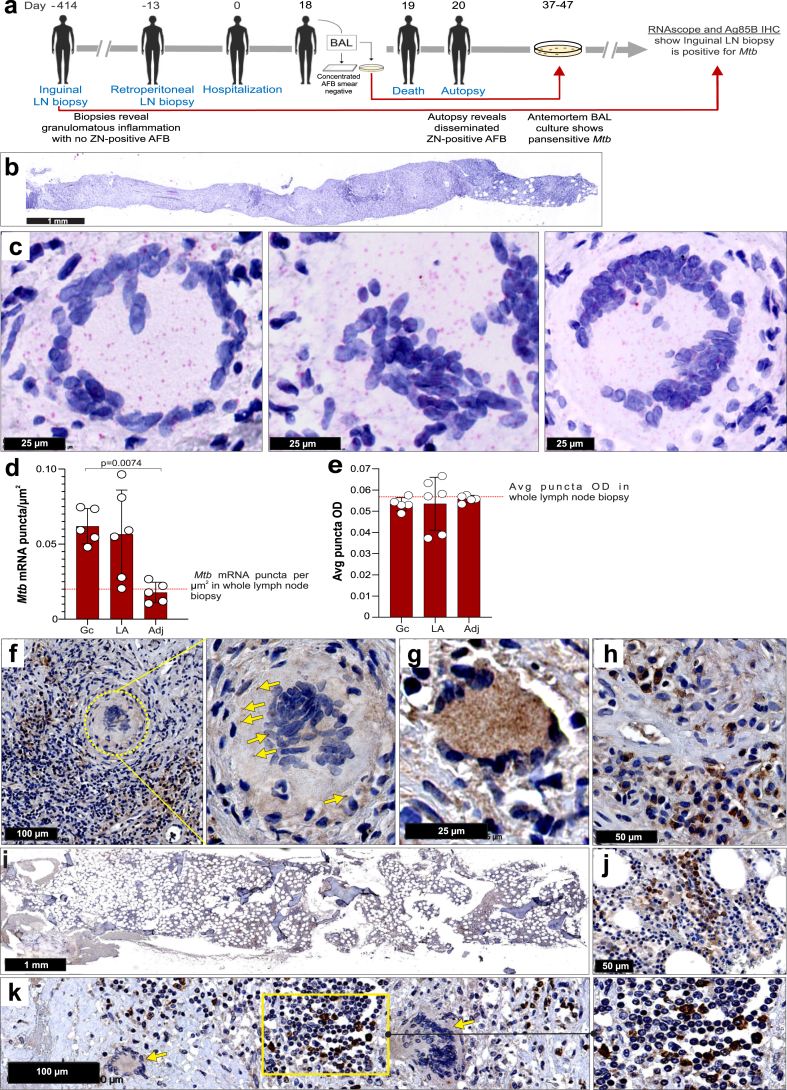
**Application of RNAscope may help guide therapeutic intervention**. (**a**) Flowchart depicting CT-guided biopsies, hospitalisation, and postmortem analyses of tissues from a patient with undiagnosed TB. (**b**) Medium power image of a ZN-negative antemortem left inguinal lymph node needle biopsy specimen obtained 414 days prior to hospital admission. (**c**) High power images of the inguinal lymph node in (**b**) with RNAscope *Mtb* mRNA puncta within and around giant cells. (**d**) Number of *Mtb* mRNA puncta per square micron. Horizontal red line indicates the average number of puncta per square micron across the entire lymph node specimen. (**e**) Average puncta optical density (OD) in and around giant cells (Gc), lymphocytic aggregates (LA) and adjacent (Adj) lymphoid tissue. Horizontal red line indicates the average OD of puncta across the entire lymph node specimen. (**f**) Ag85B staining in and around giant cells in the ZN-negative inguinal lymph node specimen. The circled area shows weakly positive giant cells with several Ag85B-positive bacilli (yellow arrows). (**g**) High power image of a strongly Ag85B-positive giant cell in the inguinal lymph node specimen. (**h**) High power image of Ag85B accumulation within the cytoplasm of lymphocytes in the inguinal lymph node specimen. (**i**) Low power image of Ag85B-positive cells in the ZN-negative antemortem bone marrow biopsy. (**j**) Medium power image showing Ag85B positivity in the cytoplasm of lymphocytes within bone marrow shown in (**i**). (**k**) Ag85B positivity in the ZN-positive postmortem periaortic lymph node specimen. Data in (**d**) and (**e**) represent the mean ± SD and each data point represents the number of *Mtb* mRNA puncta per square micron (**d**) or average puncta OD (**e**) in each zone (Gc, LA, and Adj) of the inguinal lymph node (n = 5–6). Data in (**d**) were analyzed using one-way ANOVA and Bonferroni's multiple comparison test.

To evaluate the potential of RNAscope to guide clinical intervention, we examined tissue specimens from a 61-year-old female initially diagnosed with disseminated histoplasmosis. Biopsies of inguinal and retroperitoneal lymph nodes were performed 414 and 13 days before hospitalisation, respectively (Fig. 7a). Both biopsy specimens were ZN-negative and revealed granulomatous inflammation (Supplemental Figure S7). On hospital day 18, bronchoscopy with bronchoalveolar lavage (BAL) showed a negative gram stain and AFB smear. The patient died on hospital day 19 with the cause of death attributed to complications of sepsis. Autopsy revealed disseminated granulomatous inflammation of the lung, liver, bone marrow, and periaortic lymph node (Supplemental Figure S8a-d). ZN staining showed diffuse involvement by AFB (Supplemental Figure S9), and the cause of death was amended to be complications from disseminated TB. Culture of antemortem BAL fluid revealed the presence of pansensitive *Mtb* complex 47 days after hospital admission (Fig. 7a).

With longitudinal specimens available, we subjected the ZN-negative inguinal lymph node biopsy obtained 414 days prior to hospital admission (Fig. 7b) to RNAscope analysis and found numerous *Mtb* mRNA transcripts (Fig. 7c). Quantitation of *Mtb* mRNA puncta using HALO® analysis showed a higher number of puncta around and inside giant cells and lymphocytic aggregates compared to adjacent lymphoid tissue and the whole biopsy (Fig. 7d) with no difference in average puncta optical density (OD) (Fig. 7e). This suggests that the number of *Mtb* mRNA puncta can help predict pathophysiological abnormalities in human TB tissue.

The detection of *Mtb* via RNAscope is further supported by strong Ag85B positivity in giant cells (Fig. 7f and g) and surrounding lymphocytes (Fig. 7h) in the antemortem inguinal lymph node specimen. ZN-negative antemortem bone marrow biopsy (Fig. 7i and j) and ZN-positive postmortem periaortic lymph node (Fig. 7k) specimens also demonstrated Ag85B positivity.

In this clinical scenario, application of RNAscope detection of *Mtb* mRNA and Ag85 IHC to biopsy tissue could have positively identified *Mtb*, providing much earlier diagnosis of disseminated TB, possibly enabling effective TB treatment. These findings suggest that detection of *Mtb* mRNA and antigens has diagnostic value in complex TB cases where sputum or biopsy material is ZN-negative.

## Discussion

Major unmet needs in the TB field are the ability to consistently identify *Mtb* bacilli and the means to gain a clear understanding of how *Mtb* contributes to human tissue pathology. We have taken initial steps toward addressing these needs by adapting the RNAscope platform to identify intact and disintegrating *Mtb* bacilli and single *Mtb* transcripts in human TB tissues. We show that *Mtb* mRNA puncta are highly abundant in ZN-negative lung tissue specimens from a confirmed pulmonary TB case, that *Mtb* mRNA is found intracellularly and extracellularly, and that *Mtb* mRNA and antigens accumulate in cells from histologically normal and abnormal tissue. We also provide evidence of two phenotypically distinct *Mtb* cell populations *in vivo*. Lastly, analysis of an antemortem biopsy provides evidence that RNAscope can help guide therapeutic intervention in TB cases where small biopsy material is ZN-negative. Overall, the ability of RNAscope to detect *Mtb* bacilli in diverse morphological states and identify molecular remnants *in vivo* advances our understanding of TB pathophysiology and diagnosis.

RNAscope has diagnostic promise due to its highly sensitive and specific probe design. In particular, the detection of clearly-defined bacillary rod shapes that are visually similar to positive ZN staining as well as puncta indicating single transcripts makes it an attractive diagnostic platform. Current dogma asserts that bacterial mRNA is an unlikely detection marker due to its susceptibility to degradation. Like other bacteria, *Mtb* employs a multienzyme RNA degradome comprised of endo- and exoribonucleases, an RNA helicase, and a polynucleotide phosphorylase that controls mRNA decay rates during growth and in response to environmental cues.^65^^,^^66^ While this degradome effectively degrades intrabacillary mRNA, very little is known about extrabacillary stability of *Mtb* mRNA within host cells, which is likely influenced by the specific cellular compartment (*e.g*., phagosome, phagolysosome, or cytoplasm) in which it is contained. As RNAscope is effective in detecting intra- and extracellular *Mtb* mRNA, our findings suggest that mRNA released from the bacterial cell is sufficiently stable to be detected by this platform.

Pathologists are frequently confronted with diagnostic challenges in TB cases since most human necrotic granulomas contain few, if any bacilli. This is consistent with comprehensive human pathology studies conducted by Canetti^12^ that suggest the formation of necrotic granulomas stems not from bacillary replication, but from bacillary destruction accompanied by exaggerated inflammatory responses induced by *Mtb* antigens. Subsequent studies in animal models affirmed that TB is primarily an outcome of persistent host responses to bacillary products rather than bacillary proliferation.^67^ Since RNAscope detects *Mtb* mRNA in AFB-negative lung and lymph node specimens from patients with confirmed TB, this technique could assist pathologists in confirming TB diagnosis to guide treating physicians. Furthermore, the interventional value of RNAscope was demonstrated by confirming the presence of *Mtb* mRNA in a ZN-negative antemortem biopsy from a patient initially diagnosed with histoplasmosis. Hence, RNAscope analysis could have guided potentially life-saving therapy.

*Mtb* is highly adaptive to changing host immune signaling, the onset of hypoxia, variable nutrient availability, and distinct lesion microenvironments,^68^^,^^69^ and therefore bacilli exist in diverse phenotypic states *in vivo*. However, there are few methods to identify specific phenotypes in TB tissue.^62^^,^^70^ *Mtb* naturally exists in fully, partially, and non-acid fast states and loses its acid fastness during disintegration.^12^^,^^13^ Hence, acid fastness is not an accurate indicator of viability. Several studies have shown that some *Mtb* surface and/or secreted antigens can be detected in human sputum^24^ and in human^9^^,^^17^^,^^34^^,^^64^^,^^71^ and guinea pig^72^ tissue specimens. We identified two distinct populations of *Mtb* in human specimens: those that produce Ag85B and those that do not. Since many of these bacilli are in close proximity, differential *ag85B* expression in response to different environmental signals is unlikely. Since Ag85B secretion is not universal, Ag85B IHC alone is likely to underestimate bacillary burden, consistent with prior studies.^28^

An unexpected discovery was the accumulation of *Mtb* mRNA in the cytoplasm of HAMR cells. This important finding is reminiscent of studies that demonstrated the presence of *Mtb* DNA in host cells.^51^^,^^73^ We were unable to identify discernible bacilli inside these cells, suggesting that the source of mRNA is disintegrated intracellular bacilli. An alternative explanation is that HAMR cells take up exogenous *Mtb* mRNA and/or secreted antigens. Recent studies have shown that *Mtb* can produce extracellular vesicles (EVs) that contain hundreds of proteins, including Ag85A,^74^ secrete RNA *in vitro*^75^ and release mRNA into the cytosol in macrophages.^60^ Further, EVs from *Mtb*-infected macrophages contain *Mtb* mRNA.^60^^,^^76^ Hence, uptake of *Mtb*-derived EVs by HAMR cells could account for the accumulation of mRNA and Ag85B. RNA from *Streptococcus* *agalactiae* (group B streptococcus) has been implicated as an immunomodulatory PAMP that induces IFN-β production in dendritic cells,^57^ suggesting that *Mtb* nucleic acids and/or secreted antigens may exert similar effects. Indeed, *Mtb* RNA is sensed in a manner dependent on melanoma differentiation factor 5 (MDA-5), an RNA sensor in the RIG-I-like 3 receptor family, resulting in increased IL-1β production, inflammasome activation and attenuation of autophagy. Notably, these effects culminate in increased *Mtb* survival in macrophages.^59^ Overall, these studies suggest mechanisms by which HAMR cells in patients with TB may accumulate *Mtb* mRNA and/or secreted antigens and provide context for investigating the fate and immunomodulatory function of these cells.

Our finding of *Mtb* mRNA in bronchiolar epithelial cells provides new insight into *Mtb* tropism, as this microenvironment is not routinely examined by pathologists. The accumulation of *Mtb* mRNA inside bronchiolar epithelium indicates significant bacillary destruction in this environment, which was confirmed by Ag85B positivity, and is consistent with our finding that *Mtb* can cross the bronchial epithelium.^61^

We have demonstrated the potential of RNAscope to detect *Mtb* mRNA within intact bacilli, disintegrating bacilli and single *Mtb* transcripts in a range of human tissues including pulmonary, lymphatic, and testicular specimens. Our case study involving ante- and postmortem biopsy specimens provides compelling evidence that RNAscope has the potential to reduce clinical time-to-diagnosis in TB cases in which histopathologic findings can neither confirm nor rule out the presence of *Mtb*. Positive Ag85B immunostaining confirmed the RNAscope results, increasing diagnostic confidence. In cases where clinical suspicion remains high despite negative AFB staining, RNAscope has the potential to confirm or exclude the diagnosis of TB.

Our study has limitations that could affect its application. Firstly, distinguishing viable from nonviable bacilli remains to be demonstrated since the presence of intrabacillary mRNA and presence of Ag85B is not necessarily an indication of viability. Secondly, the design and synthesis of RNAscope probes is relatively expensive, which may limit its initial clinical application.

In conclusion, our findings have important implications for TB pathophysiology and diagnosis. RNAscope detected *Mtb* mRNA *in vivo*, which was previously thought to be unstable, as well as in intact and disintegrating bacilli. We also detected *Mtb* mRNA within ZN-negative human tissues obtained from pulmonary and extrapulmonary sites and found that *Mtb* mRNA accumulates within some host cells but is also present outside host cells. Additionally, we show that antigen secretion is not universal, demonstrating two diverse populations of *Mtb in vivo*. These findings imply applications that could have considerable impact, such as the early identification of individuals who are at risk of dying from TB-related causes and tracking the clinical course of disease via the historical imprints of *Mtb*.

## Contributors

Conceptualisation and Design: KN, TN, PVB, MM, AJCS. Lung tissue preparation: MM, TN, KN, KL, PVB. Pathology: TN, RLH, PVB. Histopathology: KN, TN, MM, RLH, PVB. Data integration: GW, KL, SN, AH, AJCS. Verification of underlying data: all authors. Writing initial draft: JNG, TN, AJCS. Editing: AH, JNG, SN, GW, AJCS. Final draft: All authors. Figure preparation: SN, JNG, KN, AJCS. All authors have read and approved the final version of the manuscript.

## Data sharing statement

High-resolution RNAscope and/or IHC images generated in the study are available from the corresponding author (asteyn@uab.edu) on request, or can be viewed at: https://www.ahri.org/scientist/adrie-steyn/.

## Declaration of interests

The authors have no competing interests or disclosures.

## Appendix A Supplementary data

Supplementary Figures and Tables

Reagent validation file_datasheet.pdf
