# Supplementary material for: Spatial distribution of Mycobacterium tuberculosis mRNA and secreted antigens in acid-fast negative human antemortem and resected tissue
Source: eBioMedicine. 2024 Jun 15;105:105196. doi: 10.1016/j.ebiom.2024.105196 (PMC11233921; doi:10.1016/j.ebiom.2024.105196)
Supplement: Supplementary Figures and Tables [file mmc1.pdf]

Appendix A

Supplemental Data

Spatial distribution of *Mycobacterium tuberculosis* mRNA and secreted antigens in acid-fast negative human antemortem and resected tissue

Kievershen Nargan, Joel N Glasgow, Sajid Nadeem, Threnesan Naidoo, Gordon Wells, Robert L Hunter, Anneka Hutton, Kapongo Lumamba, Mpumelelo Msimang, Paul V Benson, and Adrie JC Steyn

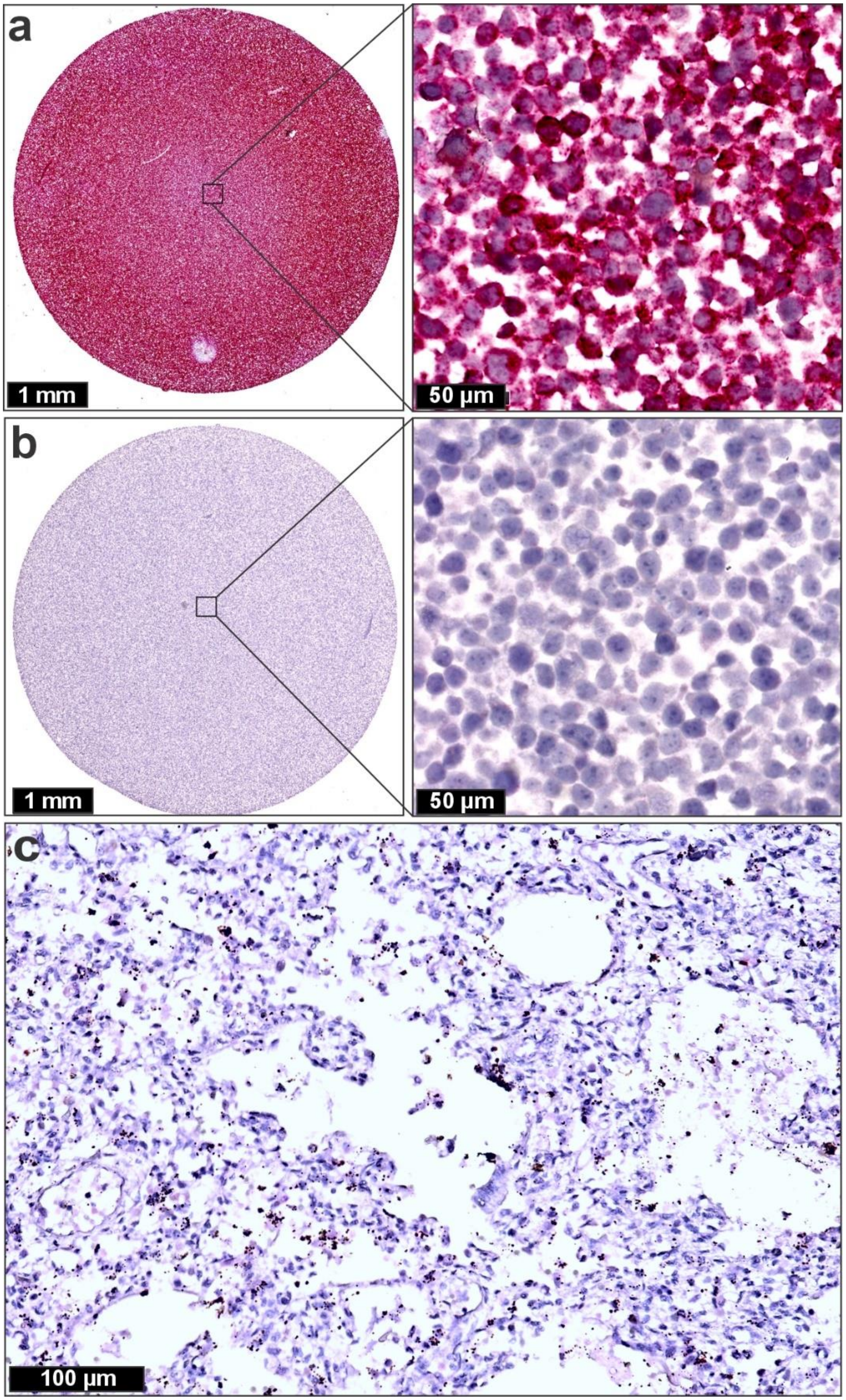

Figure S1. RNAscope probe set and tissue control assays. (**a**) Section of a Hela cell pellet exposed to the positive control RNAscope probe set directed towards human peptidylprolyl isomerase B (*PPIB*) mRNA showing strong positive signals. (**b**) Hela cell pellet section exposed to the negative control probe set specific for *Bacillus subtilis* dihydrodipicolinate reductase (*dapB*) mRNA is negative for RNAscope signals. (**c**) Human neonatal lung tissue exposed to the *Mtb*-specific probe set is negative for RNAscope signals.

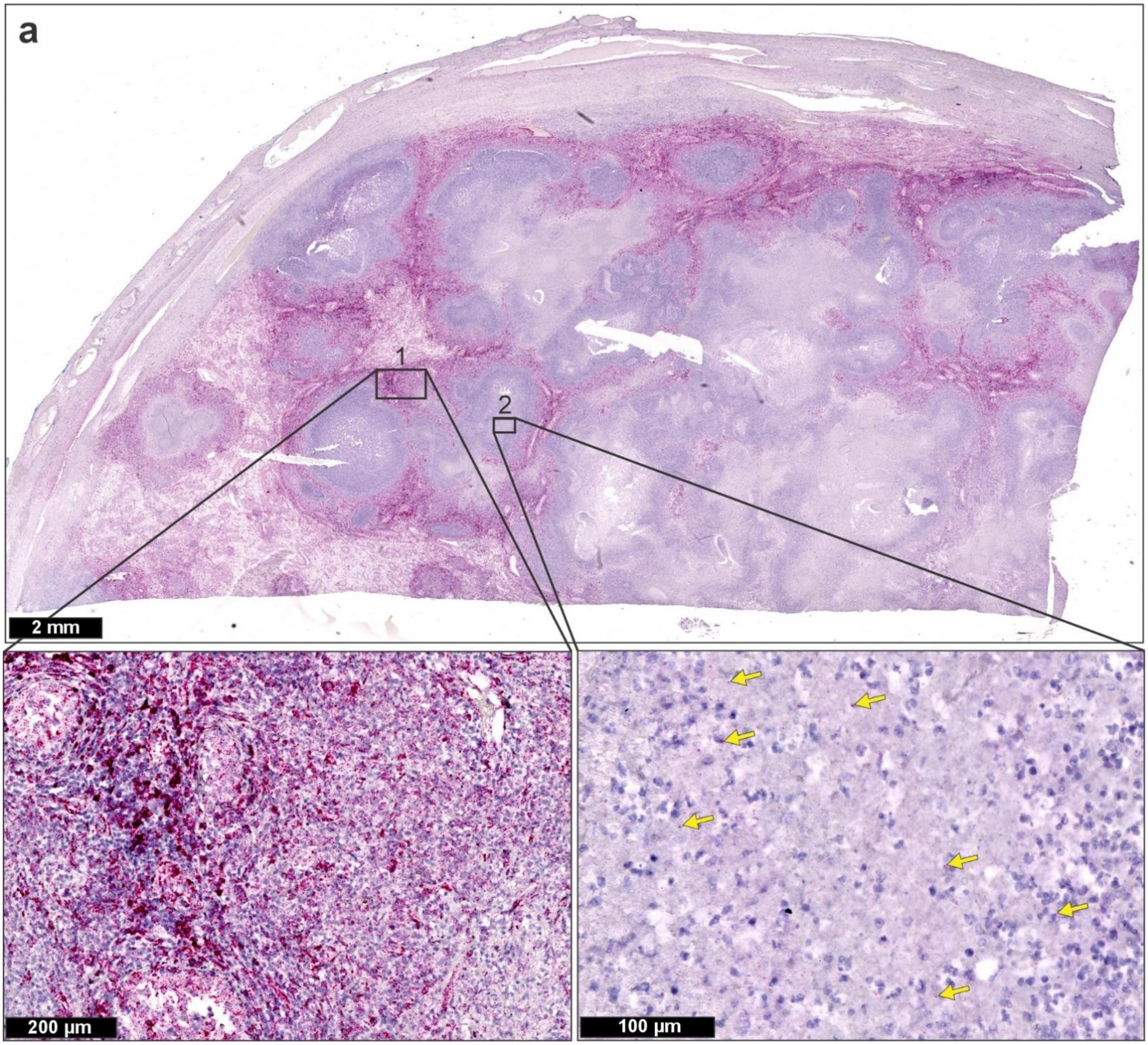

Figure S2. RNAscope tissue and probe set control assays. (**a**) Low power image of RNAscope signals from a positive control probe set directed against human peptidylprolyl isomerase B (*PPIB*) mRNA in a testicular specimen from a patient with TB. Insets; medium power images of boxed areas in (**a**). (**Box 1**) Medium power image showing an abundance of RNAscope positive (*PPIB*) signals. (**Box 2**) Yellow arrows indicate sparse RNAscope signals in the necrotic area.

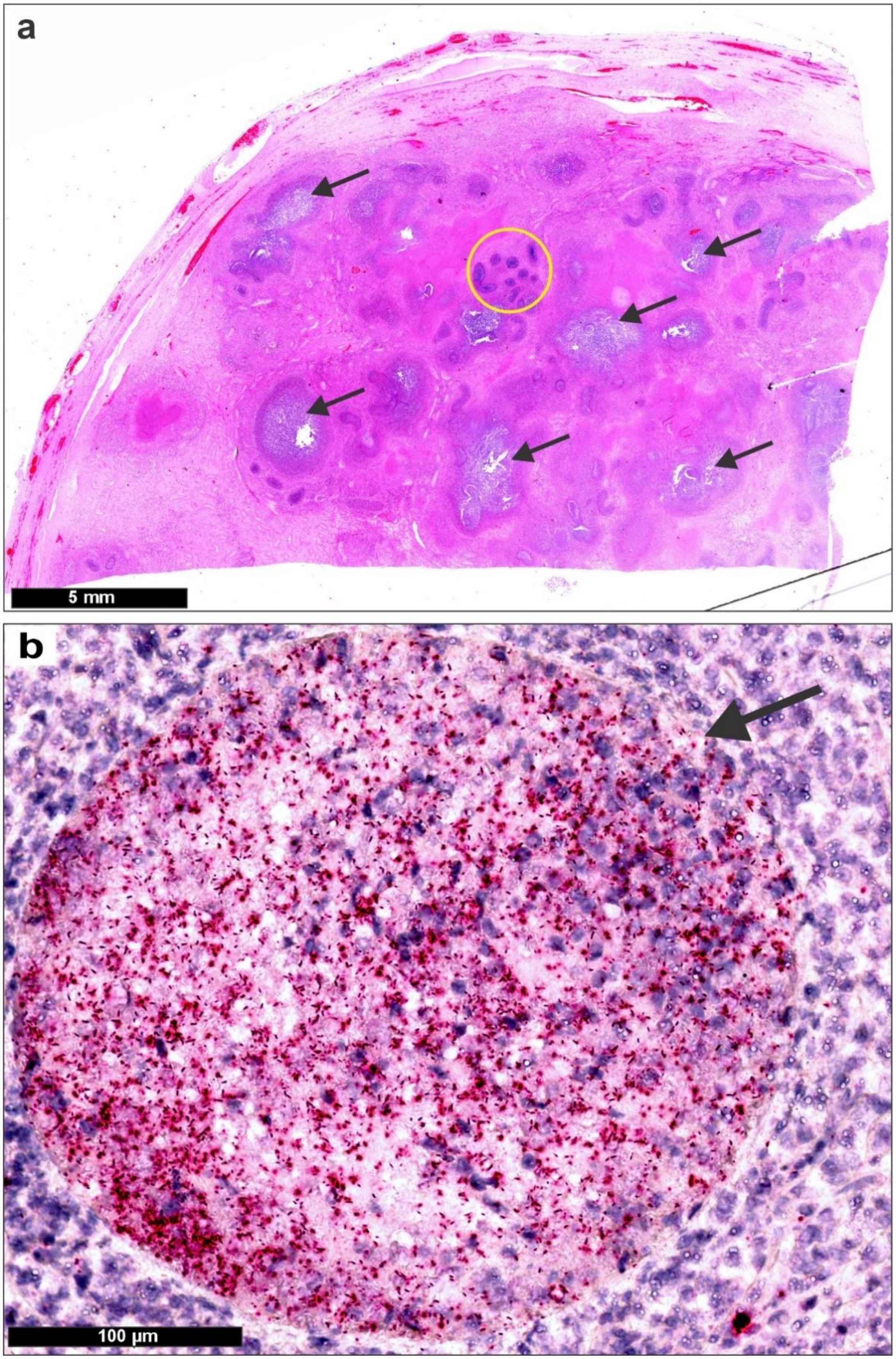

Figure S3. RNAscope detects *Mtb* in seminiferous tubules. (**a**) Low power image of an H&E-stained testicular specimen obtained from a patient with TB. Circled area; seminiferous tubules. Arrows indicate granuloma. (**b**) Medium power image of a single seminiferous tubule containing numerous *Mtb*-specific *Mtb* RNAscope signals. Black arrow indicates the tubule basement membrane.

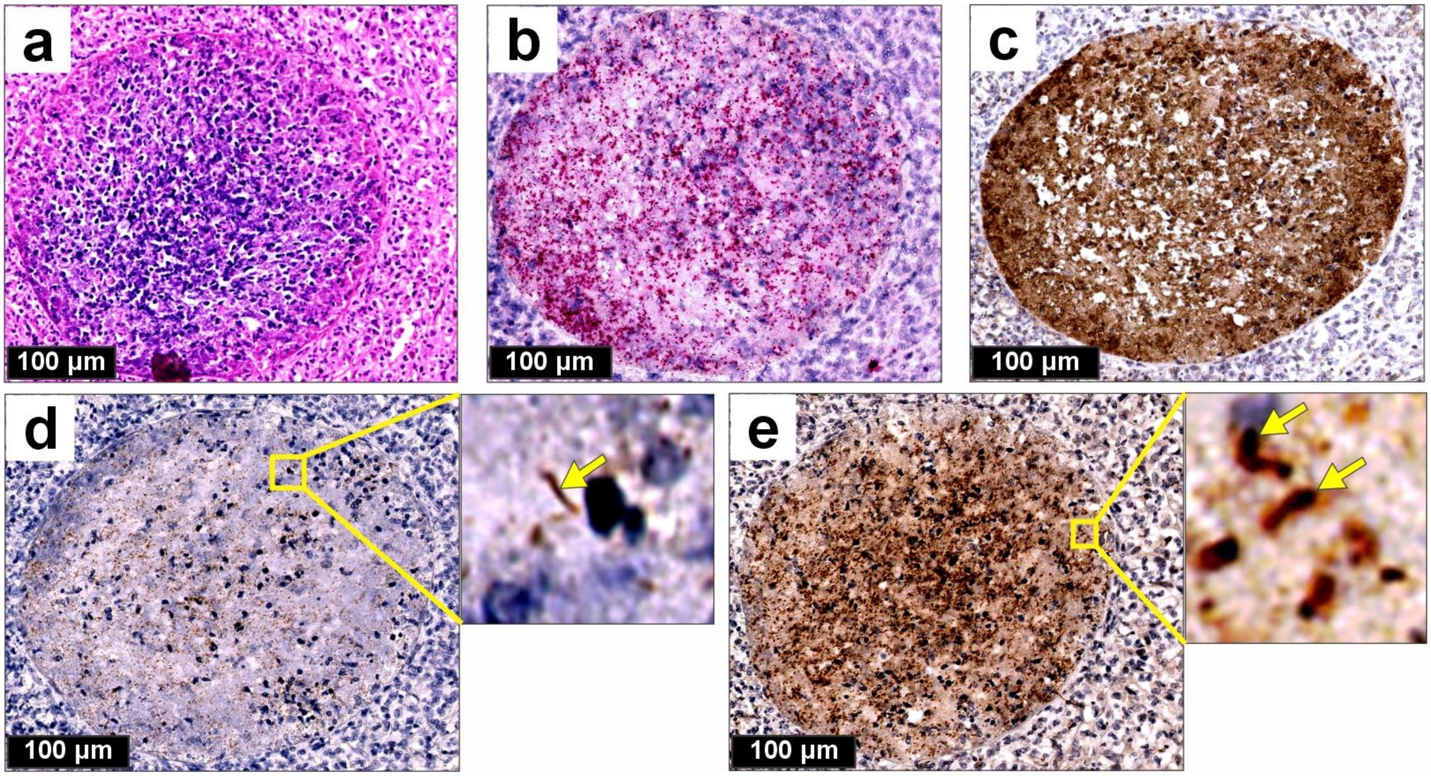

Figure S4. Accumulation of *Mtb* antigens in extrapulmonary TB tissue. Images of consecutive sections of a seminiferous tubule from a patient with TB. (**a**) Medium power image of H&E staining. (**b**) Medium power image showing *Mtb*-specific RNAscope signals (Note: same image as Figure S3b). (**c**) Medium power image of *Mtb* USP-positive IHC staining. (**d**) Medium power image of ESAT-6-positive IHC staining with a high-power image depicting ESAT-6-positive bacillary shapes (yellow arrow). (**e**) Medium power image of Ag85B-positive IHC staining with high power image of Ag85B-positive bacillary shapes (yellow arrows). (**c**, **d**, **e**) Note the clear line of demarcation formed by the tubule basement membrane between the accumulated secreted antigens inside the seminiferous tubule and the surrounding tissue confirming the specificity of the antibodies.

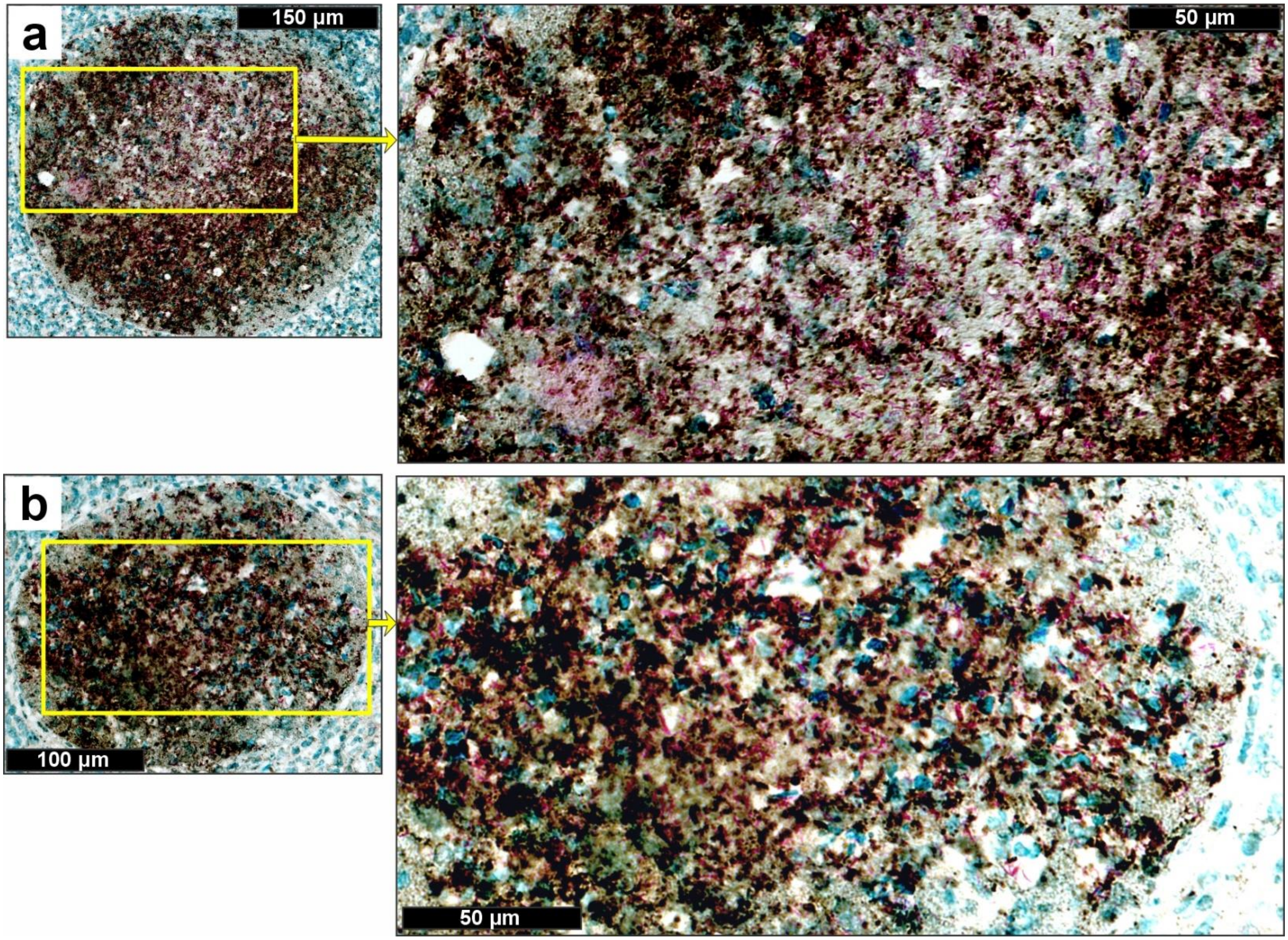

Figure S5. *Mtb* Ag85B-positive and -negative bacilli inside seminiferous tubules. (**a**) and (**b**) low power images of seminiferous tubules from a patient with TB with combined ZN staining (pink) and Ag85B IHC staining (brown). Insets; medium power images illustrating Ag85B-positive and ZN-positive/Ag85B-negative bacilli. Note: image in (**a**) is the same image shown in Figure 6a.

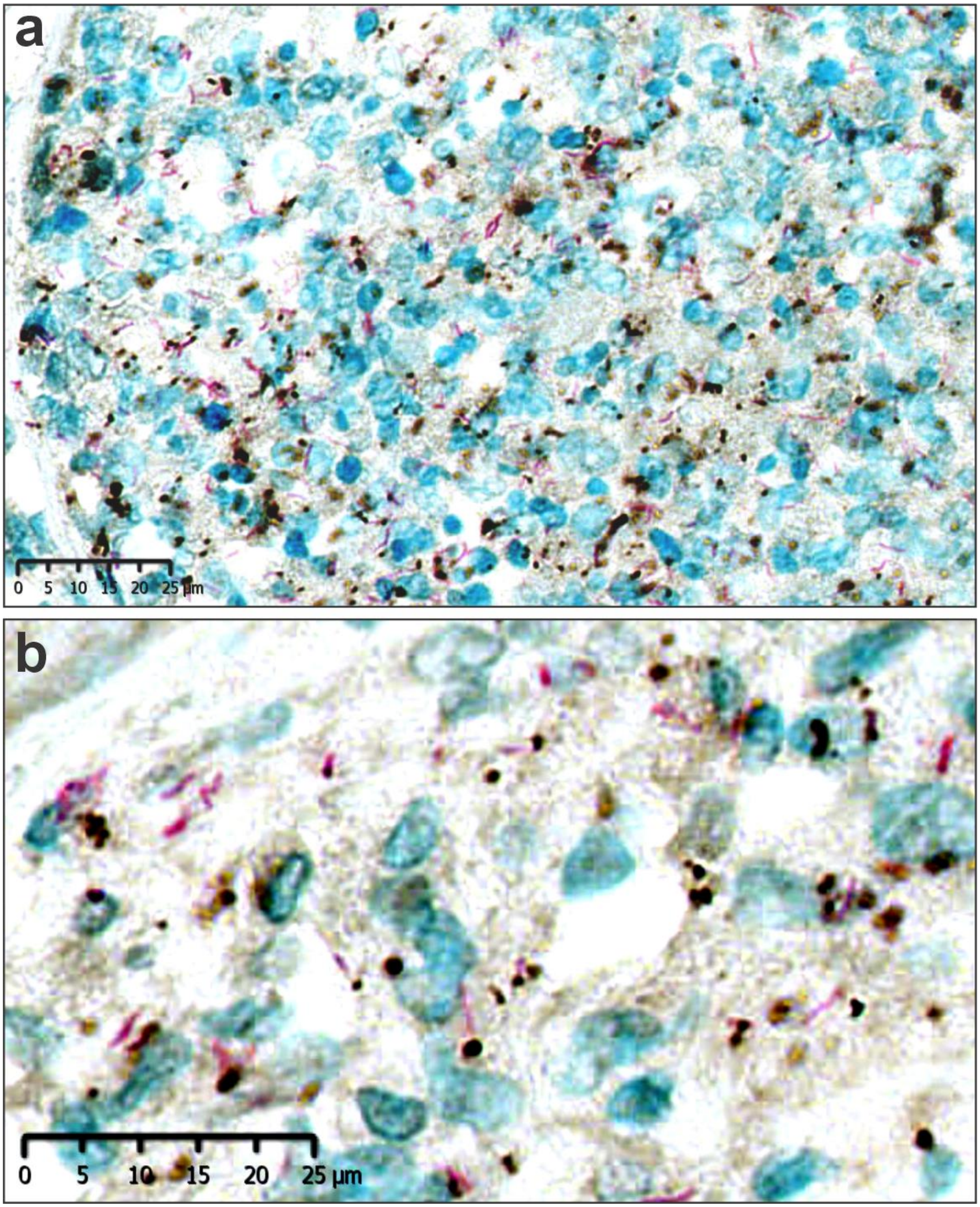

Figure S6. Ag85B-positive and -negative *Mtb* bacilli in resected extrapulmonary TB tissue. (**A**) Low power and (**B**) medium power images of testicular tissue (outside the seminiferous vesicle) with combined ZN staining (pink) and Ag85B IHC staining (brown).

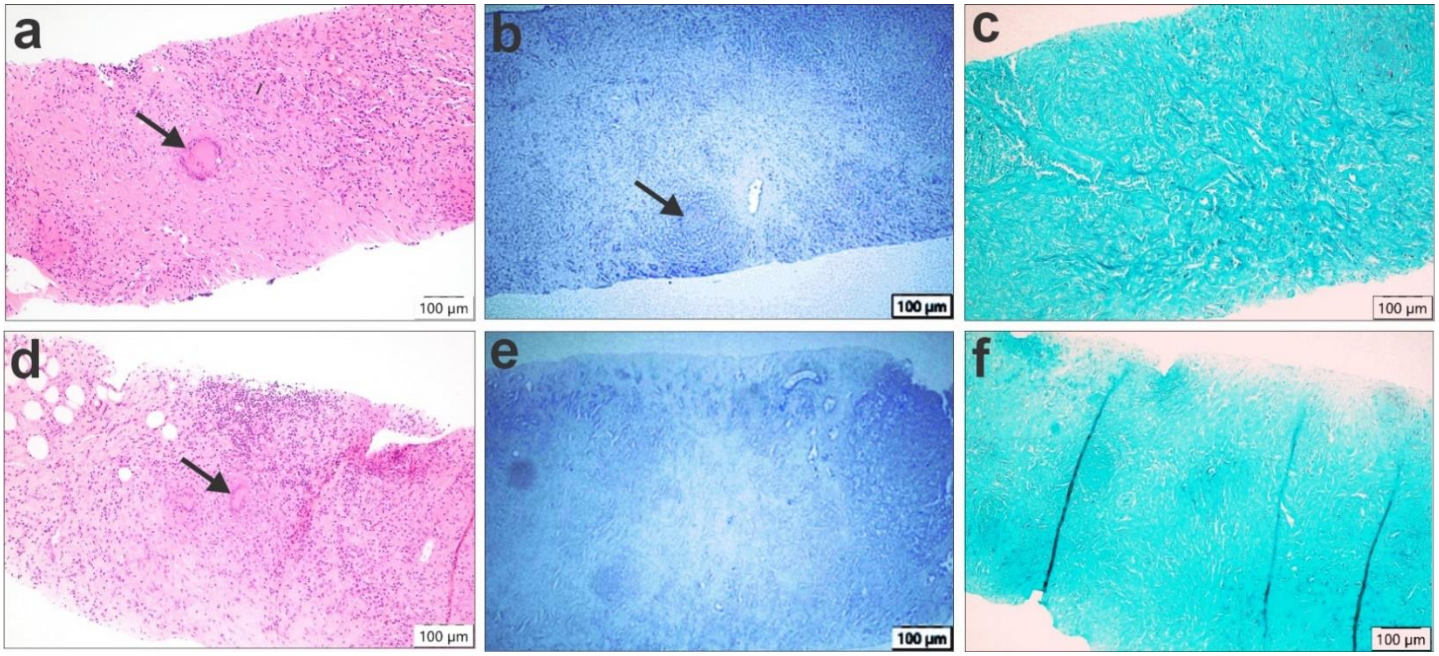

Figure S7. Antemortem biopsy specimens showing evidence of granulomatous inflammation but absence of AFB and fungal organisms. (**a**-**c**) Left inguinal lymph node biopsy collected 414 days prior to hospital admission. (**d**-**f**) Retroperitoneal lymph node biopsy collected 13 days prior to hospital admission. (**a** and **d**) H&E staining. (**b** and **e**) ZN staining shows no evidence of AFB. (**c** and **f**) Grocott-Gomori Methenamine Silver (GMS) staining shows no evidence of fungus or yeast. Black arrows indicate giant cells associated with granuloma.

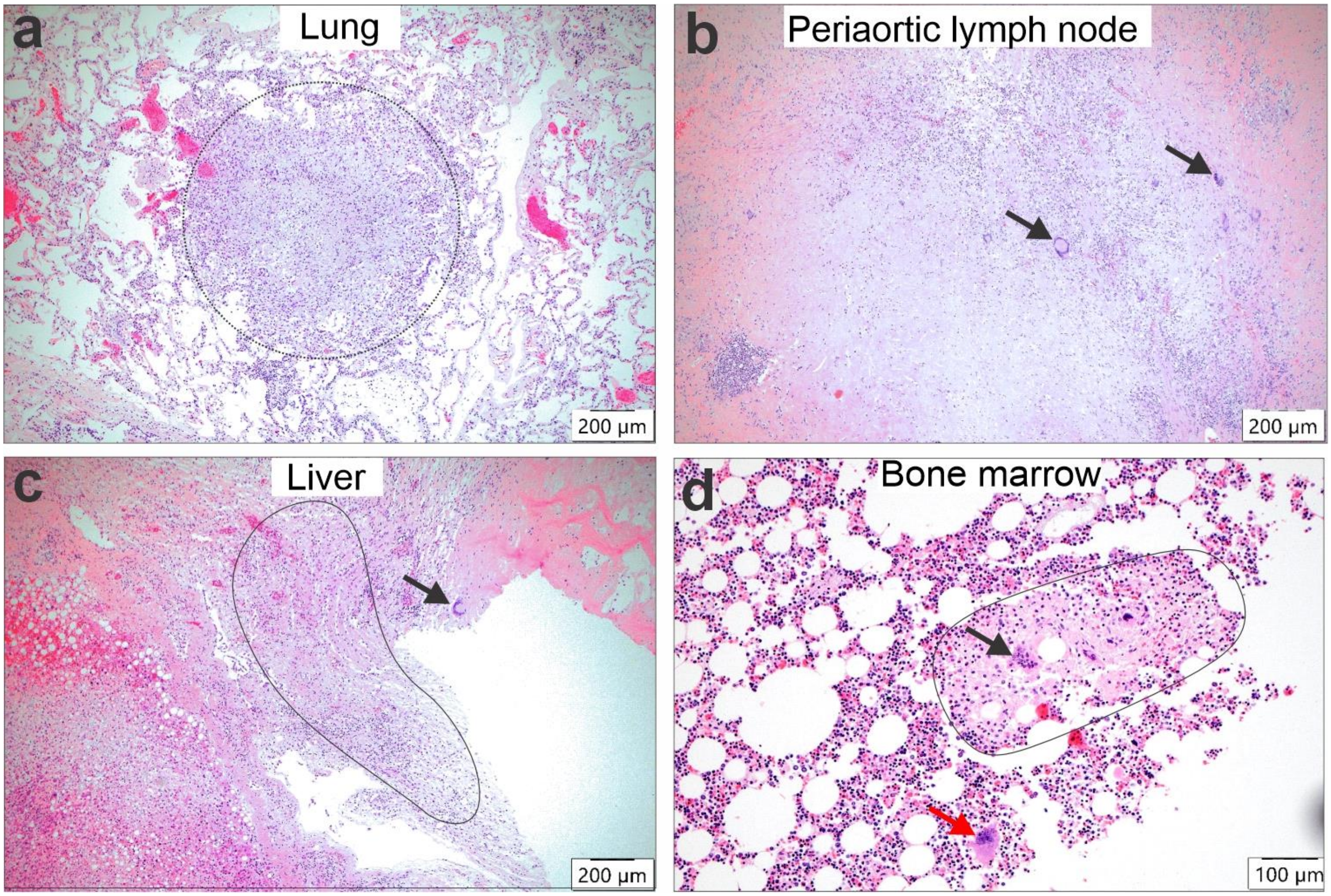

Figure S8. Autopsy specimens showing diffuse granulomatous inflammation in multiple tissues. H&E staining of (**a**) Active granuloma with necrosis in the lung (circled). (**b**) Periaortic abdominal lymph node with giant cells (black arrows). (**c**) Liver specimen with necrotic fibrin exudate (circled) and giant cell (black arrow). (**d**) Organized granuloma (circled) in bone marrow with giant cell (black arrow). Red arrow, megakaryocyte.

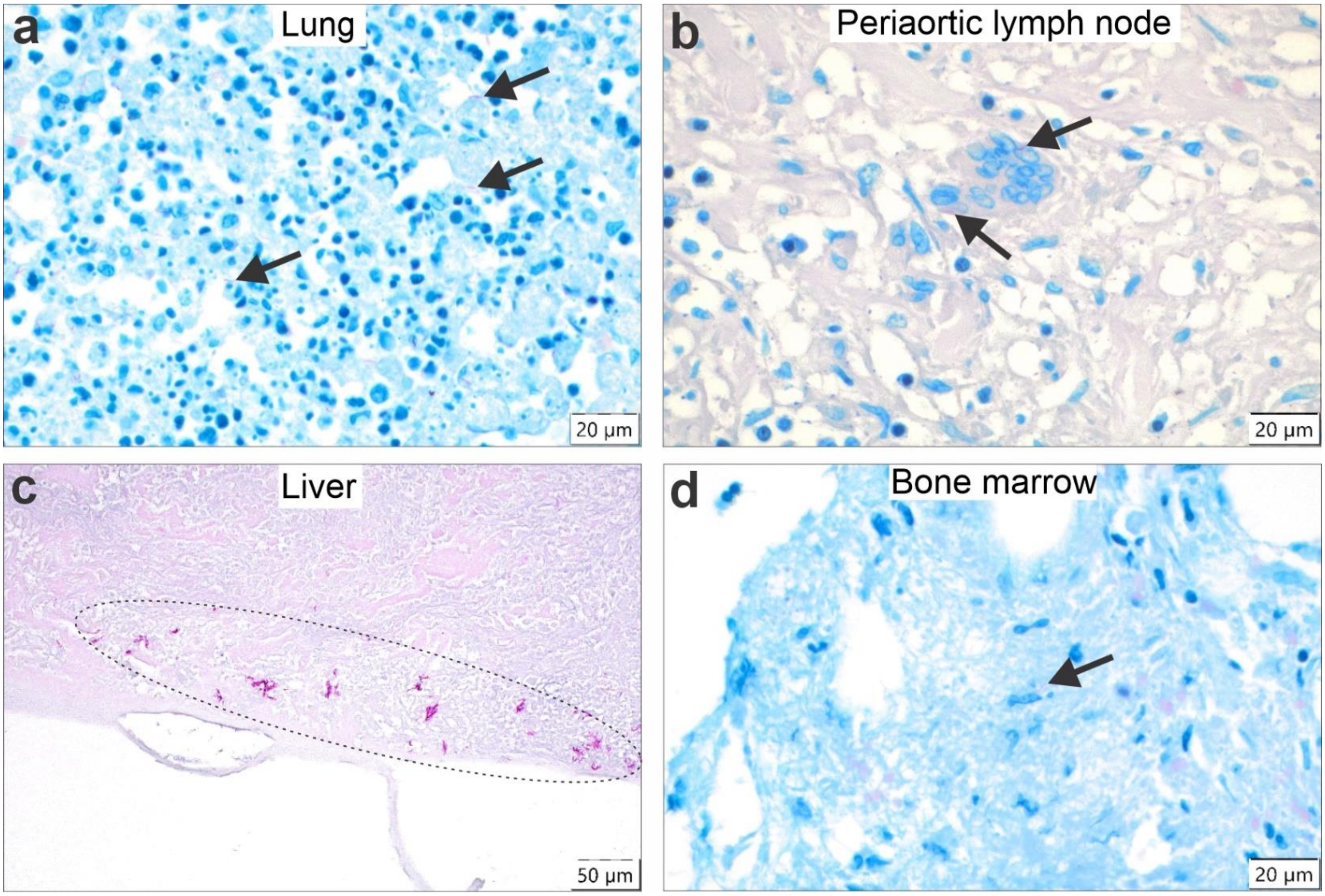

Figure S9. Autopsy specimens showing acid fast bacilli in multiple tissues. ZN staining of autopsy specimens from (**a**) Lung, (**b**) Periaortic abdominal lymph node, (**c**) Liver, and (**d**) Bone marrow. Black arrows and area within the oval indicate ZN-positive *Mtb* bacilli. ZN stain includes methylene blue counter stain. Liver section displayed surface-localized necrotic fibrin exudate that prevented nuclear staining.

Table S1. Clinical characteristics of human subjects

Patient ID #	Age	Sex	HIV	Tissue specimen	Macroscopic and microscopic features
SL314-23	67	M	Pos	Lobectomy, left upper lobe	Lobe weighing 323 g, pleural surface was hemorrhagic with patchy areas of caseous necrosis. Pathology features are those of necrotizing granulomatous inflammation with AFB, consistent with active TB.
SL463-23	38	F	Pos	Lobectomy, left lung	Lobe weighing 281 g, cut sections showed evidence of bronchiectasis and caseative necrosis, a cavity was noted. Areas of necrotizing granulomatous inflammation were evident, and AFB were identified. Suppurative granulomas with microabscess formation were noted.
SL114-23	30	F	Pos	Pneumonectomy, left Lung	Left lung weighing 229 g, lung was shrunken, fibrotic and cavitated with miliary tuberculosis. Numerous AFB were present, features of lymphoid interstitial pneumonia were present.
SL038-23	38	M	Pos	Left orchidectomy	Left testis weighing 108 g, upon cut section, architectural distortion by extensive necrotizing granulomatous inflammation was demonstrated. Granulomas showed central caseative necrosis, blood vessels demonstrate extensive medial hypertrophy with luminal occlusion. Numerous AFBs were identified. Features are those of tuberculous epididymo-orchitis.
SLPM1-23		F	Neg	Neonatal lung tissue	Death shortly after birth and prior to administration of BCG vaccination. Routine postmortem lung samples (minimum 1 section from each lobe of the right and left lungs). Normal anatomy with no gross pulmonary pathology. Microscopy revealed variable alveolar expansion with patchy intra-alveolar oedema and hemorrhage. Infectious pathogens, granulomata or neoplastic infiltrates were not seen.
21-106	61	F	Neg	Antemortem: Inguinal lymph node, retroperitoneal lymph node, bone marrow.<br><br>Postmortem: Lungs, periaortic abdominal lymph node, liver, and bone marrow.	Antemortem lymph nodes biopsies revealed granulomatous inflammation on H&E and were negative for AFB on ZN. Antemortem bone marrow revealed focal paratrabecular plasma cells and was not ZN stained clinically due to no granulomas present.<br><br>Postmortem lung tissue, periaortic abdominal lymph node, liver, and bone marrow all revealed diffuse granulomatous inflammation and were positive for AFB on ZN staining. See clinical imaging notes below*.

*A chest X-ray 27 days prior to hospitalization showed no obvious lymphadenopathy with unremarkable heart and lungs.

A chest/abdomen/pelvis CT on hospital day 1 showed no evidence of acute aortic syndrome or central pulmonary thromboembolism. A tree-in bud nodularity in the right middle was noted along with multiple ill-defined upper lobe nodules, suggesting possible infection or inflammation. Metastatic disease was not completely excluded. There was no evidence of retroperitoneal hematoma. Interval increase in simple ascites was noted. Hepatic steatosis was observed with recommendation to correlate with liver enzymes to exclude acute hepatitis. Heterogeneous enhancement spleen was seen, possibly related to arterial phase examination. Small splenic infarcts were not completely excluded. Spleen also appeared enlarged, increased in size from a prior exam, and follow-up was recommended. The patient exhibited right axillary, intrathoracic and extensive intra-abdominal lymphadenopathy.

Due to dyspnea, a chest X-ray on hospital day 5 was performed revealing normal heart and clear lung. Tree-in-bud opacities in the small upper lobe nodules were not identified but may have been too small to see. No acute disease or evidence of pneumonia was noted.

A chest X-ray on the day of death (hospital day 19) showed small layering bilateral pleural effusions, right greater than left and hazy bilateral opacities, right greater than left persist. No pneumothorax was seen with stable cardiomediastinal silhouette.

Culturing of tissue biopsy material was not performed. Antemortem BAL fluid was negative for AFB, but cultures were later found to be positive for *Mtb*. Urine LAM tests were not performed. Other clinical data include a positive urine histoplasma antigen test on hospital day 14. Seven days prior to hospitalization, a T-spot test gave a borderline result and ACE levels were elevated (152 units/L).

#Patient identity cannot be determined from these anonymized ID numbers

Table S2. Parameters and values for RNAscope signal quantitation using the HALO software platform

Parameters	Values
**Stain Selection**	
Number of Probes	1
Set the nuclear stain	0.644, 0.716, 0.267
Probe Name	RNA Probe
Set the RNA Probe stain	0.242, 1.151, 0.452
Set the exclusion stain	2.407, 2.407, 2.407
Output Image	RNA Probe Markup
**Optimize spot/signal detection**	
RNA Probe Markup Color	255, 0, 0
RNA Probe Contrast Threshold	0.02
RNA Probe Minimum Optical Density	0.053
Spot Segmentation Aggressiveness	0.95
RNA Probe Spot Size	0.8, 20
RNA Probe Size	2.1 or 1.8
Fill RNA Probe Holes	True
Output Image	RNA Probe Markup
**Set Exclusion Marker**	
Exclusion Threshold	0.2
Exclusion Radius	0.5
