# Supplementary material for: Spatial distribution of Mycobacterium tuberculosis mRNA and secreted antigens in acid-fast negative human antemortem and resected tissue
Source: eBioMedicine. 2024 Jun 15;105:105196. doi: 10.1016/j.ebiom.2024.105196 (PMC11233921; doi:10.1016/j.ebiom.2024.105196)
Supplement: Reagent validation file_datasheet.pdf [file mmc2.pdf]

abcam

Product datasheet

Anti-Mycobacterium tuberculosis Ag85B antibody ab43019

★★★★☆ 4 Abreviews 18 References 2 Images

Overview

**Product name**	Anti-Mycobacterium tuberculosis Ag85B antibody
**Description**	Rabbit polyclonal to Mycobacterium tuberculosis Ag85B
**Host species**	Rabbit
**Specificity**	This antibody is specific for the Ag85B protein in sera, plasma or cell culture supernatant.
**Tested applications**	**Suitable for:** IHC-P, WB, ELISA
**Species reactivity**	**Reacts with:** Mycobacterium tuberculosis
**Immunogen**	Recombinant full length protein (Mycobacterium tuberculosis)
**General notes**	The Life Science industry has been in the grips of a reproducibility crisis for a number of years. Abcam is leading the way in addressing this with our range of recombinant monoclonal antibodies and knockout edited cell lines for gold-standard validation. Please check that this product meets your needs before purchasing.<br>If you have any questions, special requirements or concerns, please send us an inquiry and/or contact our Support team ahead of purchase. Recommended alternatives for this product can be found below, along with publications, customer reviews and Q&As

Properties

**Form**	Liquid
**Storage instructions**	Shipped at 4°C. Upon delivery aliquot and store at -20°C. Avoid freeze / thaw cycles.
**Storage buffer**	Constituents: 0.05% Glycerol (glycerin, glycerine), 0.6057% Tris, 1.2114% Sodium citrate
**Purity**	Protein A purified
**Clonality**	Polyclonal
**Isotype**	IgG

Applications

The Abpromise guarantee Our **Abpromise guarantee** covers the use of ab43019 in the following tested applications.

The application notes include recommended starting dilutions; optimal dilutions/concentrations should be determined by the end user.

Application	Abreviews	Notes
IHC-P	★★★★☆ (1)	Use at an assay dependent concentration.
WB	★★★☆☆ (2)	1/3000 - 1/8000. Predicted molecular weight: 34 kDa.
ELISA	★★★★★ (1)	1/5000 - 1/10000.

Target

Relevance

Antigen 85B is the most abundant protein expressed by Mycobacterium tuberculosis (about one quarter). It is a myocolyc transferase in the myc pathway and catalyses - like Ag85A and Ag85C - the transfer of the fatty acid mycolate from one trehalose monomycolate to another, resulting in trehalose dimycolate and free trehalose and helping build the cell wall.

Images

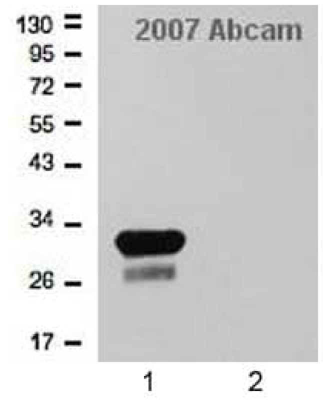

Western blot - Anti-Mycobacterium tuberculosis Ag85B antibody (ab43019)

This image is courtesy of an anonymous Abreview

All lanes : Anti-Mycobacterium tuberculosis Ag85B antibody (ab43019) at 1/5000 dilution

All lanes : E coli, expressing his-tagged Ag85b

Secondary

Lane 1 : HRP conjugated goat anti-rabbit IgG

Lane 2 : HRP conjugated goat anti-rat IgG

Predicted band size: 34 kDa

Observed band size: 34 kDa

Additional bands at: 28 kDa (possible non-specific binding), 28 kDa (possible degradation product)

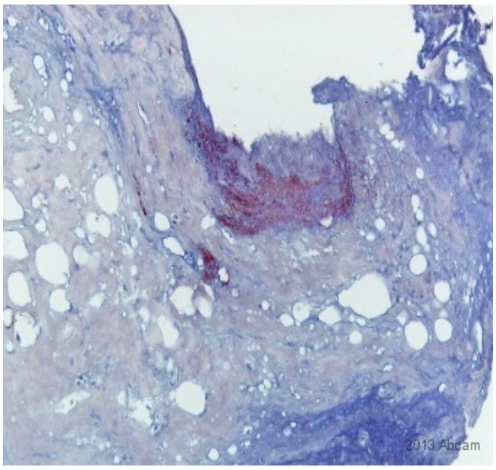

Immunohistochemistry (Formalin/PFA-fixed paraffin-embedded sections) - Anti-Mycobacterium tuberculosis Ag85B antibody (ab43019)

This image is courtesy of an anonymous Abreview

ab17976 staining Mycobacterium tuberculosis Ag85B in Human lung tissue sections by Immunohistochemistry (IHC-P - paraformaldehyde-fixed, paraffin-embedded sections). Tissue was fixed with formaldehyde. Samples were incubated with primary antibody (1/100 in antibody diluent) for 1 hour at 25°C. A HRP-conjugated Goat anti-rabbit IgG polyclonal was used as the secondary antibody.

Please note: All products are "FOR RESEARCH USE ONLY. NOT FOR USE IN DIAGNOSTIC PROCEDURES"

Our Abpromise to you: Quality guaranteed and expert technical support

- Replacement or refund for products not performing as stated on the datasheet
- Valid for 12 months from date of delivery
- Response to your inquiry within 24 hours

- We provide support in Chinese, English, French, German, Japanese and Spanish
- Extensive multi-media technical resources to help you
- We investigate all quality concerns to ensure our products perform to the highest standards

If the product does not perform as described on this datasheet, we will offer a refund or replacement. For full details of the Abpromise, please visit **https://www.abcam.com/abpromise** or contact our technical team.

Terms and conditions

- Guarantee only valid for products bought direct from Abcam or one of our authorized distributors

abcam

Product datasheet

Anti-ESAT6 antibody ab45073

3 Abreviews 4 References

Overview

**Product name**	Anti-ESAT6 antibody
**Description**	Rabbit polyclonal to ESAT6
**Host species**	Rabbit
**Specificity**	The rabbit polyclonal antibody to EsaT-6 recognizes the EsaT-6 (Rv3875) protein of Mycobacterium tuberculosis and Mycobacterium bovis.
**Tested applications**	**Suitable for:** WB
**Species reactivity**	**Reacts with:** Mycobacterium bovis, Mycobacterium tuberculosis
**Immunogen**	Recombinant full length protein corresponding to ESAT6.
**General notes**	The Life Science industry has been in the grips of a reproducibility crisis for a number of years. Abcam is leading the way in addressing this with our range of recombinant monoclonal antibodies and knockout edited cell lines for gold-standard validation. Please check that this product meets your needs before purchasing.<br>If you have any questions, special requirements or concerns, please send us an inquiry and/or contact our Support team ahead of purchase. Recommended alternatives for this product can be found below, along with publications, customer reviews and Q&As

Properties

**Form**	Liquid
**Storage instructions**	Shipped at 4°C. Store at +4°C short term (1-2 weeks). Upon delivery aliquot. Store at -20°C long term.
**Storage buffer**	pH: 7.40<br>Preservative: 0.097% Sodium azide<br>Constituent: PBS
**Purity**	Protein A purified
**Clonality**	Polyclonal
**Isotype**	IgG

Applications

The Abpromise guarantee Our **Abpromise guarantee** covers the use of ab45073 in the following tested applications.

The application notes include recommended starting dilutions; optimal dilutions/concentrations should be determined by the end user.

Application	Abreviews	Notes
**WB**	★☆☆☆☆ (2)	Use a concentration of 0.5 - 1 µg/ml. Predicted molecular weight: 11 kDa. Use under reducing conditions.

Target

Relevance The ESAT6 antigen from Mycobacterium tuberculosis is a dominant target for cell mediated immunity in the early phase of tuberculosis (TB) in TB patients as well as in various animal models. ESAT6 is not found in M. bovis BCG. Its function is not known but it elicits high level of IFN-gamma from memory effector cells during the first phase of a protective immune response.

Cellular localization Secreted

Please note: All products are "FOR RESEARCH USE ONLY. NOT FOR USE IN DIAGNOSTIC PROCEDURES"

Our Abpromise to you: Quality guaranteed and expert technical support

- Replacement or refund for products not performing as stated on the datasheet
- Valid for 12 months from date of delivery
- Response to your inquiry within 24 hours

- We provide support in Chinese, English, French, German, Japanese and Spanish
- Extensive multi-media technical resources to help you
- We investigate all quality concerns to ensure our products perform to the highest standards

If the product does not perform as described on this datasheet, we will offer a refund or replacement. For full details of the Abpromise, please visit **https://www.abcam.com/abpromise** or contact our technical team.

Terms and conditions

- Guarantee only valid for products bought direct from Abcam or one of our authorized distributors

LSBio
an Absolute Biotech Company

Enter catalog no. or keywords, e.g. cd70, lag3, hmgb1 elisa

Antibodies > Mycobacterium tuberculosis Uncharacterized Protein > LS-C683286

Catalog Number	Size	Price
LS-C683286-500	500 µl (5 mg/ml)	$363

Specifications

Description

Mycobacterium tuberculosis Uncharacterized Protein antibody LS-C683286 is an unconjugated rabbit polyclonal antibody to mycobacterium tuberculosis Mycobacterium tuberculosis Uncharacterized Protein. Validated for ELISA, IF, IHC, LateralFlow and WB.

Target

Mycobacterium tuberculosis Uncharacterized Protein
See All Mycobacterium tuberculosis Uncharacterized Protein Antibodies

Host

Rabbit

Reactivity

Mycobacterium tuberculosis (tested or 100% immunogen sequence identity)

Clonality

Polyclonal

Conjugations

Unconjugated

Purification

Greater than 95%

Modifications

Unmodified

Immunogen

Purified protein derivative (PPD) of M. tuberculosis

Specificity

M. tuberculosis

Applications

- IHC
- Immunofluorescence
- Western blot
- ELISA
- LateralFlow

Presentation

10 mM PBS, pH 7.2, 0.1% Sodium Azide

Storage

Short Term: Store at 2°C to 8°C. Long Term: At -20°C. Avoid freeze/thaw cycles.

Restrictions

For research use only. Intended for use by laboratory professionals.

Guarantee

This antibody carries the LSBio 100% Guarantee.

About Mycobacterium tuberculosis Uncharacterized Protein

NCBI: A2VL01

Publications (0)

Customer Reviews (0)

Request SDS/MSDS

To request an SDS/MSDS form for this product, please contact our Technical Support department at:

Technical.Support@LSBio.com

Requested From: United States
Date Requested: 5/27/2024

Family of Companies

LSBio Nordic MUbio everest absolute antibody exalpha kerafast

Follow LSBio

View Cart

Register or Login

Copyright © 2022 LifeSpan BioSciences, Inc - Privacy Policy

Contact Us

2 Shaker Rd Suites
B001/B101
Shirley, MA 01464

Tel: +1 (206) 374-1102
Fax: +1 (206) 577-4565

To place an order:
Orders@LSBio.com

For questions about orders:
Customer.Support@LSBio.com

For technical assistance:
Technical.Support@LSBio.com
